# Discovery of Indole-Based PDE5 Inhibitors: Synthesis and Pharmacological Evaluation

**DOI:** 10.1021/acsmedchemlett.5c00108

**Published:** 2025-05-28

**Authors:** Shin-Young Park, Dang Pham, Param Shukla, Justin Edward, Reshmi John, Addison Li, Michael Hadjiargyrou, Mattia Mori, Elisa Zuccarello, Ottavio Arancio, Jole Fiorito

**Affiliations:** † Department of Biological and Chemical Sciences, 6196 New York Institute of Technology, Old Westbury, New York 11568, United States; ‡ Department of Biotechnology, Chemistry and Pharmacy, 9313 University of Siena, 53100 Siena, Italy; § Taub Institute for Research on Alzheimer’s Disease and the Aging Brain, 5798 Columbia University, New York, New York 10032, United States; ∥ Department of Medicine, Columbia University, New York, New York 10032, United States; ⊥ Department of Pathology & Cell Biology, Columbia University, New York, New York 10032, United States

**Keywords:** PDE5, PDE5 Inhibitors, Indole-containing Molecules, Alzheimer’s Disease

## Abstract

Phosphodiesterase 5 (PDE5) inhibitors have been suggested as new treatments for Alzheimer’s disease (AD) and other conditions such as cancer and cardiovascular diseases. Utilizing the widespread presence of the indole moiety in biomolecules and drugs, previously synthesized quinoline and naphthyridine compounds were modified into novel indole-containing PDE5 inhibitors. Replacing the amine with an amide group led to identifying a potent analogue, compound **14a**, with an IC_50_ of 16.11 nM. Molecular docking simulations further highlight the significance of the amide group in drug-target interactions. A cytotoxicity test and a parallel artificial membrane permeability assay validated the compound’s potential as a lead for further drug development. Compound **14a** was shown to be safe and blood-brain barrier permeable. The discovery of these indole-containing PDE5 inhibitors provides new perspectives for developing PDE5 therapeutics.

## Graphical abstract

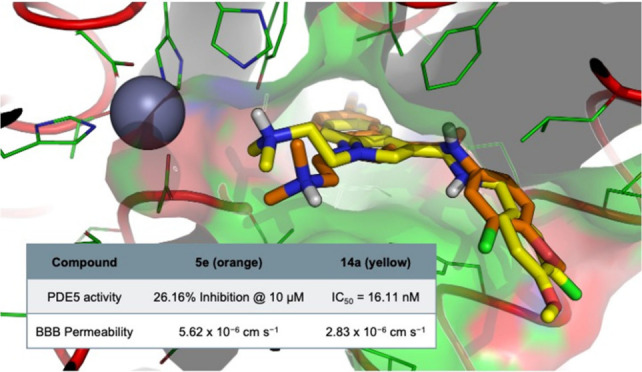

Phosphodiesterase 5 (PDE5) is a well-known enzyme that specifically regulates the degradation of the second messenger cyclic guanosine monophosphate (cGMP) into 3′,5′-guanosine monophosphate as part of a signaling pathway that maintains physiological cellular levels of cGMP through the cooperation of multiple effectors, such as nitric oxide (NO), particulate and soluble guanylyl cyclase (pGC and sGC, respectively), and cGMP-dependent protein kinase G (PKG).^1,2^ The inhibition of PDE5 leads to increased cellular levels of cGMP, ensuring the activation of intracellular signaling events. PDE5 is a relevant pharmacological target for the treatment of several diseases, including erectile dysfunction and pulmonary arterial hypertension. The Food and Drug Administration (FDA)-approved PDE5 inhibitors (PDE5Is) are sildenafil, tadalafil, vardenafil, and avanafil (Figure 1).^3–5^ Sildenafil and vardenafil are benzenesulfonamide molecules containing a heterocyclic ring similar to the guanine ring of cGMP. Tadalafil is classified as a carboline-based compound, while avanafil is a pyrimidine-5-carboxamide derivative bearing a 3-chloro-4-methoxybenzylamino group. Most recently, PDE5Is have also been investigated for the treatment of benign prostatic hyperplasia, cardiovascular diseases, and neurodegenerative diseases such as Alzheimer’s disease (AD).^6–10^

**Figure 1:**
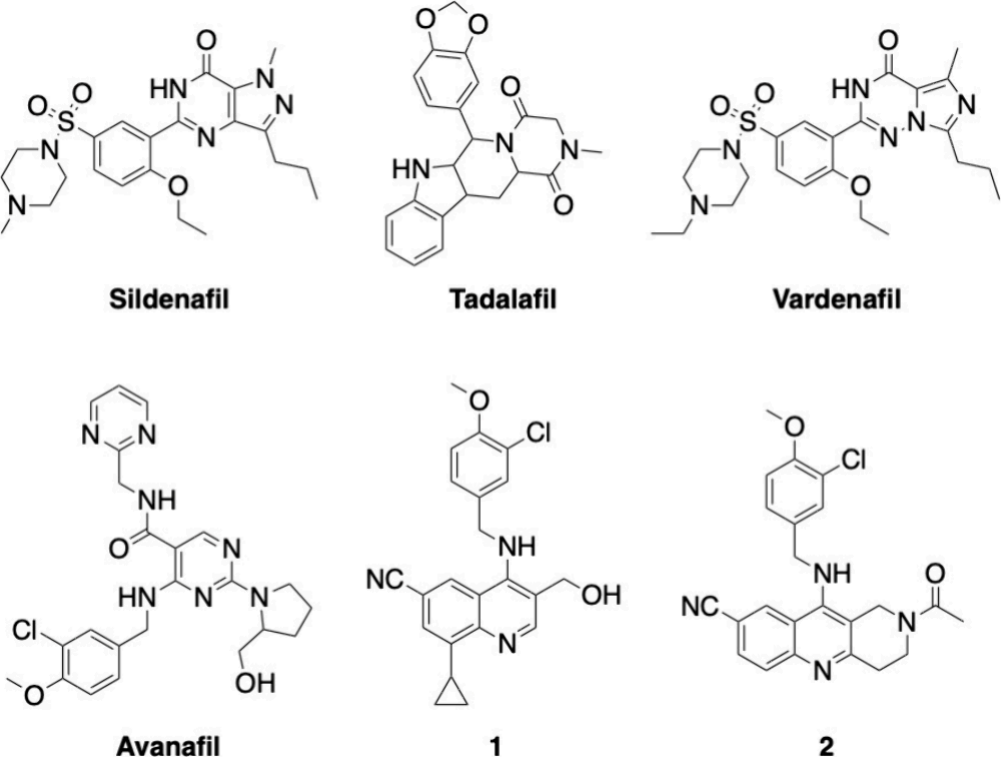
Structures of FDA-approved (sildenafil, tadalafil, vardenafil, and avanafil), quinoline-based (**1**), and naphthyridine-based (**2**) PDE5 inhibitors.

Our endeavor to identify new PDE5Is for treating neurodegenerative disorders has led to a significant breakthrough with the discovery of compounds **1** and **2** (Figure 1), which contain a quinoline and naphthyridine ring, respectively, and the 3-chloro-4-methoxybenzylamino group.^11,12^ In a computational analysis of compound **2**, we observed that the amino group establishes an H-bond with Gln817 of the PDE5 catalytic pocket, and the amide carbonyl oxygen interacts with Met816 through a water molecule. At the same time, the cyano group plays an important role in the enzymatic binding, forming an H-bond with Gln775.^12^ Moreover, a previous study demonstrated that the absence of the cyano group in a quinoline derivative with a similar structure to compound **1** is detrimental to the PDE5 activity.^13^ Compounds **1** and **2** were previously shown to function as potent PDE5Is and improve learning and memory processes in behavioral studies on AD mouse models.^11,12,14^ Expanding on our previous work, we set out to investigate the effects of the heterocyclic ring on the PDE5′s biological activity. To this end, we introduced a structural modification that reduced the ring size of the heterocyclic ring by substituting the quinoline with the indole ring, while retaining the cyano group (Figure 2).

**Figure 2:**
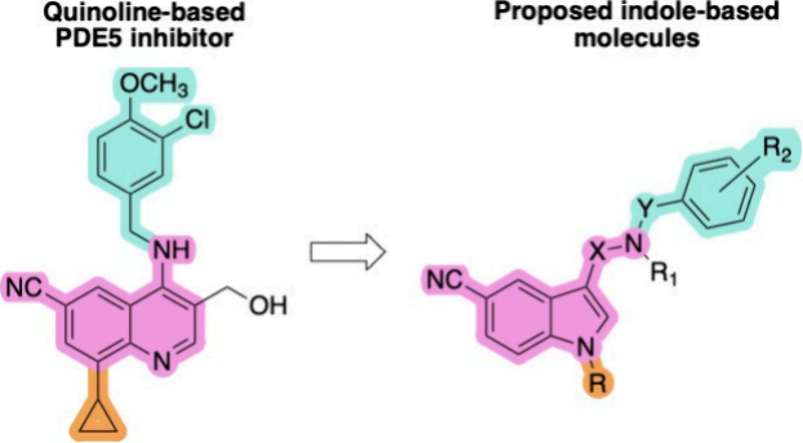
General structure of proposed indole-based molecules.

Additionally, we explored the distance between the heterocyclic and benzene rings, designing small molecules bearing a 1–4 atom nitrogen-containing linker, and moved the alkyl substituent of the quinoline ring to the indole nitrogen (Figure 2). It is worth noting that the indole ring is a key component in many natural biological molecules, including tryptophan, serotonin, and melatonin. The indole system is also found in several drugs, including the anticancer agents vincristine and vinblastine, triptans used for migraine treatment, antiemetic serotonin receptor antagonists such as ramosetron, dolasetron, and tropisetron, and the reverse transcriptase inhibitor delavirdine.^15^ Thus, discovering new, small indole-containing molecules with potential biological activity toward the PDE5 enzyme may provide lead compounds for developing novel therapeutics for several pathophysiological conditions.

In this study, we report on the synthesis and PDE5 pharmacological evaluation of new indole-containing compounds, a computational study to account for the compounds’ binding interaction in the enzymatic pocket, and a cytotoxicity evaluation and a parallel artificial membrane permeability assay (PAMPA) of the most promising compound.

First, the quinoline ring of compound **1** was replaced with an *N*-methylindole ring bearing a substituted aminomethyl group in position 3 and a cyano group in position 5, leading to the generation of a small library of compounds **5a** and **5h**–**k** (Scheme 1). We then explored the structure–activity relationship of different groups bonded to the indole nitrogen, designing compounds **5b**–**g** with careful consideration of common strategies for structural modification, e.g., chain extension and branching, structural rigidification, and substitution with hydrophobic or hydrophilic groups. Derivatives **5a**–**k** were synthesized via a simple two-step route. First, the alkylation of the indole nitrogen starting from 3-formyl-1*H*-indole-5-carbonitrile, the alkyl or benzyl halide, and NaH provided intermediate **4a**–**g**, which underwent a reductive amination reaction with NaBH_4_ in MeOH to yield the final compounds bearing different aminomethyl groups (Scheme 1).

**Scheme 1:**
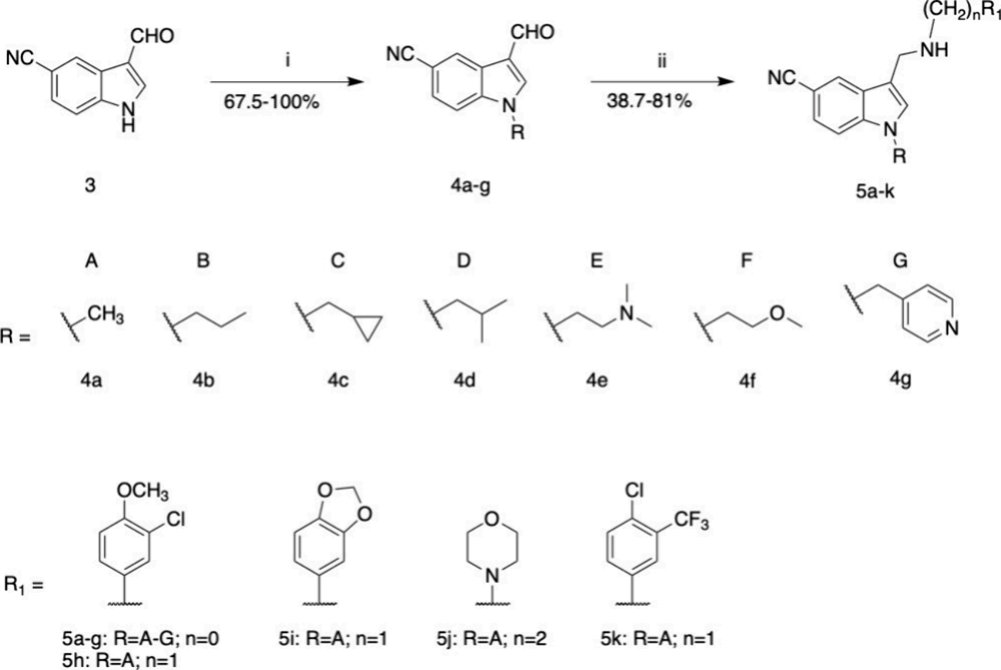
Synthesis of Compounds **5a**–**k** ^a^

a Reagents and conditions: (i) Alkyl halide, NaH, THF, rt, 3 h, or alkyl halide, Cs_2_CO_3_, 80 °C, 3–20 h; (ii) (a) substituted amine, MeOH, rt, 18–24 h, (b) NaBH_4_, rt, 1–4 h.

In addition to the 3-aminomethyl *N*-alkyl indole derivatives, we investigated *N*-tosyl indole compounds bearing a substituted aniline moiety directly linked to the indole ring in position 3. While the tosyl group was initially chosen as a protecting group for the indole nitrogen, this substituent was retained in the molecule to mimic the sulfonamide group in sildenafil and vardenafil. Compound **10** was prepared following the synthetic procedures reported in Abe *et al*. (Scheme 2).^16^ Shortly, 1*H*-indole-5-carbonitrile **3** was *N*-tosylated using *p*-toluenesulfonyl chloride in the presence of NaOH and TEBA to form intermediate **6**. The reaction between the *N*-tosyl indole **7** and NBS in water and the subsequent addition of Et_3_N resulted in the formation of **8** as a white crystalline solid. The amination reaction with 3-chloro-4-methoxyaniline provided intermediate **9**, which was dehydrated to the final compound **10** when treated with BF_3_·Et_2_O. The same synthetic route was employed to synthesize analogues of **10** with different substituents on the indole nitrogen, such as methyl and mesyl groups. However, the reaction with NBS and Et_3_N to form the alkyl aminium bromide, similar to compound **8**, was unsuccessful. Hence, we refrained from pursuing additional structural modifications on this scaffold.

**Scheme 2:**
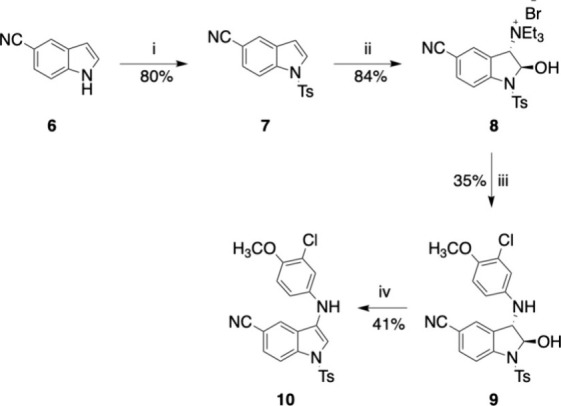
Synthesis of Compound **10** ^a^

a Reagents and conditions: (i) TsCl, TEBA, NaOH, DCM, rt, 24 h; (ii) *N*-bromosuccinimide, H_2_O, acetone, Et_3_N, rt, 2 h; (iii) aniline, Et_3_N, AcOEt, reflux, 1.5 h; (iv) BF_3_ Et_2_O, Et_3_N, AcOEt, 50 °C, overnight.

Lastly, we designed the indole derivative **14a**, in which the amino functional group was replaced with an amide (Scheme 3). Such a substitution was implemented to reduce free bond rotation in the linker due to the partial double bond character of the amide group while retaining an H-bonding donor group. Furthermore, an amide in position 3 of the indole ring mimics the amide in the naphthyridine ring of compound **2** (Figure 1), possibly providing an additional point of interaction with the enzyme. A nucleophilic substitution reaction allowed the formation of **14a** from amide **13a**, which was obtained through a classic amide coupling reaction of **12a** with 3-chloro-4-methoxybenzylamine in EDC as a coupling agent. The carboxylic acid **12a** was obtained through an oxidation reaction that used isobutylene as a chlorine scavenger,^17^ starting from the commercially available aldehyde **11a**. No unexpected or unusually high safety hazards were encountered throughout the syntheses.

**Scheme 3:**
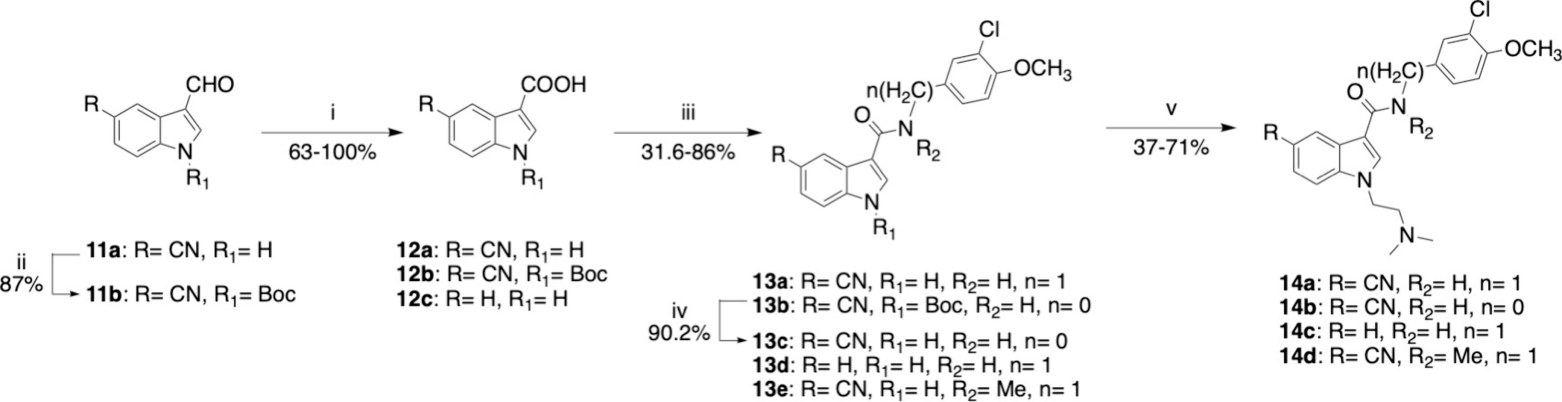
Synthesis of Compounds **14a**–**d** ^a^

a Reagents and conditions: (i) 2-methyl-2-butene, NaHPO_4_, NaClO_2_, *t*-butanol/H_2_O/MeOH, rt, 1–4 days; (ii) Boc_2_O, DMAP, DCM, rt, 24 h; (iii) amine, DMAP, DIEA, EDC, DCM, rt, or amine, PyBroP, DIPEA, DCM, 40 °C, 24 h; (iv) TFA, DCM, rt, 2.5 h; (v) 2-chloro-*N*,*N*-dimethylamine, Cs_2_CO_3_, DMF, 80 °C, overnight.

All final products were characterized by ^1^H NMR, ^13^C NMR, and HRMS (data reported in ). Moreover, the purity of these molecules was determined by liquid chromatography/mass spectrometry (LC/MS). Notably, when subjected to electrospray ionization, we observed that the *N*-alkyl indole derivatives **5a**–**k** showed a typical fragmentation pathway, which included a peak for the *N*-alkylated indole ring missing the arylamino moiety (data are summarized in Supporting Information, Table S2).

Compounds **5a**–**k**, **10**, and **14a** were tested for their ability to inhibit the PDE5 enzyme. Results of PDE5 inhibition were measured through an enzymatic assay coupled with LC/MS analysis for cGMP determination (Table 1) following a modified procedure reported by Chau *et al*.^18^ Initially, the enzymatic inhibition was measured for all compounds at a single concentration of 10 μM or 0.5 μM. IC_50_ values were calculated for compounds that showed more than 70% inhibition. Figure S1 in Supporting Information shows the concentration-percent activity curve for **14a.** Results were compared to sildenafil, which was used as a positive control to validate our experiments and generally agreed with previously published results.^19,20^ Minor discrepancies could be attributed to differences in the experimental technique. Assay details, including the LC/MS method and data analysis, are reported in Supporting Information.

**Table 1:** PDE5 Inhibition Activity of Compounds **1** and **2**, Indole Derivatives **5a-k**, **10**, and **14a-d**, and Sildenafil^a^

Compounds	% PDE5 Inhibition^b^ ± SEM	PDE5 IC_50_ (μM)^c^ ± SEM
**Sildenafil**	58.23 ± 2.33	0.0035 ^4^
**1**	N/A	0.000277 ^11^
**2**	N/A	0.000056 ^12^
**5a**	77.0 ± 4.87	2.65 ± 0.48
**5b**	94.82 ± 2.58	2.46 ± 0.13
**5c**	86.33 ± 1.97	1.37 ± 0.30
**5d**	99.24 ± 2.72	1.72 ± 0.72
**5e**	26.16 ± 3.85	N/A
**5f**	74.54 ± 1.95	10.36 ± 0.15
**5g**	54.45 ± 4.21	N/A
**5h**	72.2 ± 1.25	1.25 ± 0.15
**5i**	23.6 ± 2.24	N/A
**5j**	10.5 ± 1.69	N/A
**5k**	73.41 ± 4.13	1.77 ± 0.39
**10**	73.43 ± 7.50	N/A
**14a**	101.13 ± 4.99	0.0161 ± 0.007
**14b**	47.54 ± 2.33	10.60 ± 1.99
**14c**	66.14 ± 2.34	3.65 ± 0.97
**14d**	52.00 ± 6.68	4.90 ± 0.68

a Results are the average of at least three independent experiments.

b Sildenafil and **14a** were tested at 10 nM and 0.5 μM, respectively. All other compounds were tested at 10 μM.

c Compound concentration range was 0.1 nM – 100 μM. The substrate concentration was 150 nM.

None of the indole analogues bearing the 3-((3-chloro-4-methoxyphenyl)­amino)­methyl moiety and different alkyl groups at the indole nitrogen (**5a**–**g**) showed a potent inhibitory effect on PDE5, scoring similar IC_50_ values in the low micromolar range. These results suggest that these compounds, regardless of the alkyl group attached to the indole nitrogen, fail to engage the enzymatic binding site successfully. Additionally, changing the 3-((3-chloro-4-methoxyphenyl)­amino)­methyl portion with various aryl groups (**5h**–**k**) resulted in no improvement of potency. Interestingly, when the amine group was replaced with an amide group (compound **14a**), we observed an unexpected 2-log unit increase in biological activity (Table 1). Finally, the low percentage inhibition of the *N*-tosyl derivative **10** indicates that neither the presence of an arylsulfonyl group on the indole nitrogen nor the shortening of the amine chain between the indole ring and the aryl one provides an improved affinity for the enzymatic binding site.

Molecular modeling studies were carried out to elucidate the binding mode of the active compounds in the catalytic site of PDE5. To this aim, **5e** and **14a** were selected due to their remarkably different results in the PDE5 inhibition assay despite their chemical similarity.

First, we analyzed the protonation state of the molecules with the MoKa program, which returned a p*K*_a_ of 8.79 and 7.96 for **5e** and **14a**, respectively.^21^ This suggests that the protonated form of both molecules is predominant in aqueous buffers, although in experimental conditions, a residual neutral form of **14a** could coexist with the predominant protonated form.

Molecular docking simulations were then carried out using a protocol adapted from our previous work.^12^ The X-ray crystallography structure of PDE5 in complex with a selective inhibitor (PDB-ID: 3TGG) was used as a rigid receptor in docking with the GOLD program, using the ChemPLP docking and scoring function.^22,23^ The reliability of the docking protocol was also preliminarily assessed by redocking the crystallographic inhibitor, obtaining a docking pose with a root-mean-square deviation (RMSD) lower than 1.00 Å compared to the crystallographic pose (data not shown).

In analogy with previous findings, docking results show that **5e** can adopt two main poses within the PDE5 catalytic site, which share a π-π stacking interaction with Phe820, while they differ in the position of the nitrile group within the PDE5 site, which is docked (i) toward the solvent area or (ii) in the inner part of the catalytic site in proximity to Gln775. In contrast, docking of **14a** unequivocally identified the pose (ii) having the nitrile group near Gln775 (Figure 3A,B, and Figure S2 in Supporting Information).

**Figure 3:**
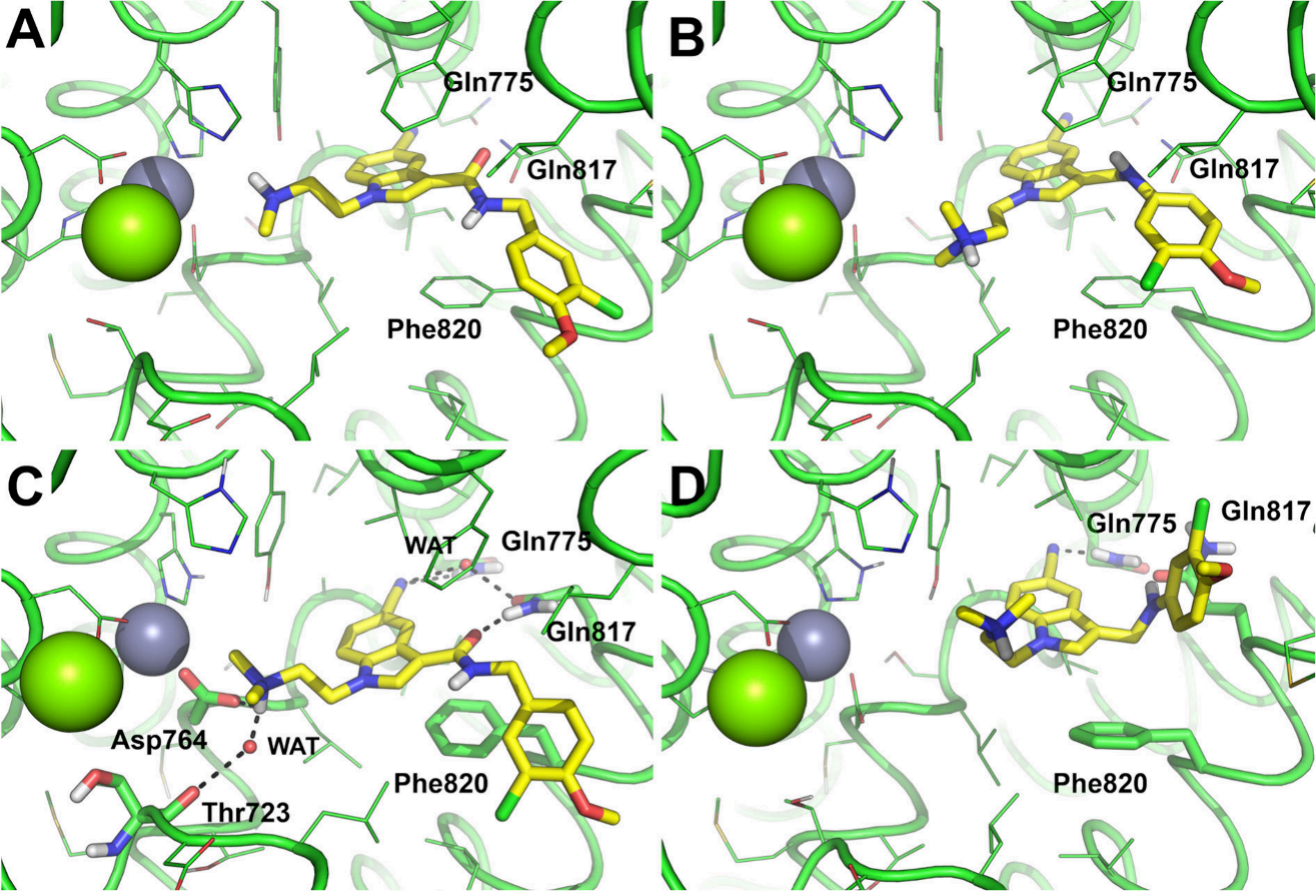
Binding mode of **5e** and **14a** to PDE5 as predicted by molecular docking and MD simulations. (A and B) Docking poses of **14a** (A) and **5e** (B) as predicted by the GOLD docking program toward the X-ray crystallography structure of PDE5 (PDB-ID: 3TGG). Both compounds are shown in the conformation with the nitrile group in the inner part of the PDE5 site (yellow stick poses). Key residues highlighted by molecular docking are shown as sticks and are labeled. Metal cations are shown as gray (Zn) and green (Mg) spheres. Residues within 4 Å from the ligands are shown as lines. (C and D) Representative frames extracted from MD trajectories run on docking complexes between PDE5 and **14a** (C) and **5e** (D). Ligands are shown as yellow sticks, H-bond interactions are highlighted by black dashed lines, residues contacted by the ligands are shown as sticks and are labeled. Water molecules bridging H-bonds are shown in panel C as small red spheres and are labeled as WAT. Metal cations are shown as gray (Zn) and green (Mg) spheres. Residues within 4 Å from the ligands are shown as lines. For clarity, all images in panels A–D were generated with the same orientation of PDE5 upon structural alignment, nonpolar H atoms are omitted.

However, the dimensionless docking scores for the two compounds in the comparable pose of Figure 3A,B are similar, failing to explain the different bioactivity profiles observed experimentally. Instead, the docking data suggested that the pose of **5e** endowed with the nitrile group in the inner part of the PDE5 site might be more affine than the pose with the nitrile group toward the solvent area at the site’s entrance (Table 2). To elucidate the structural determinants that may account for the differing bioactivity of **5e** and **14a**, molecular dynamics (MD) simulations were conducted for 500 ns, starting from the docking complexes. Notably, the **5e** pose having the nitrile group projected toward the solvent area proved unstable in MD simulations, as the molecule changed its orientation to adopt a pose in which the nitrile group is H-bonded to Gln775 within the inner part of the PDE5 site (Figure 3D). Similarly, docking poses of **14a** and **5e**, having the nitrile group already within the inner part of the active site, proved stable in MD simulations. As such, MD results converged toward a unique binding mode of **5e** and **14a** within the PDE5 site that is characterized by common interactions, including a parallel-displaced π-π stacking with Phe820 and an H-bond to Gln775 (Figure 3C,D). In addition to these interactions, which are exploited by several crystallographic PDE5 inhibitors,^24–27^ **14a** establishes further interactions with PDE5 that might explain its stronger inhibitory potency than **5e**. Specifically, **14a** can stably bind the Zn­(II) coordinating Asp764 by a charge-assisted H-bond, as well as it is H-bonded through a bridging water molecule (WAT, Figure 2C) to Thr723. The nitrile group of **14a** establishes an additional water-bridged H-bond with Gln817 (Figure 3C), while an H-bond with the side chain of Gln817, a key residue in binding to PDE5 inhibitors, further reinforces **14a** binding to the PDE5 site. The interaction network was also confirmed by a pharmacophoric analysis carried out with LigandScout on two representative frames extracted by MD trajectories through frame cluster analysis (Figures 4A and 4B).^28^ To further speculate on the different interaction schemes of **5e** and **14a** within the PED5 site, the delta energy of binding was calculated along MD trajectories by the molecular mechanics generalized Born surface area (MM-GBSA) approach.^30^ Results clearly show that **14a** is endowed with a stronger interaction energy to PDE5 (MM-GBSA delta energy of binding = – 58.22 kcal/mol) compared to its analogue **5e** (MM-GBSA delta energy of binding = – 47.57 kcal/mol), in agreement with experimental results. It is worth mentioning that the same network of interaction and comparable delta energy of binding were observed by MD simulations for the neutral form of **14a** (MM-GBSA delta energy of binding = – 53.71 kcal/mol), indicating that the protonation state might not impair the ability of **14a** to fit the PDE5 catalytic site (Supporting Information, Figure S3).

**Figure 4:**
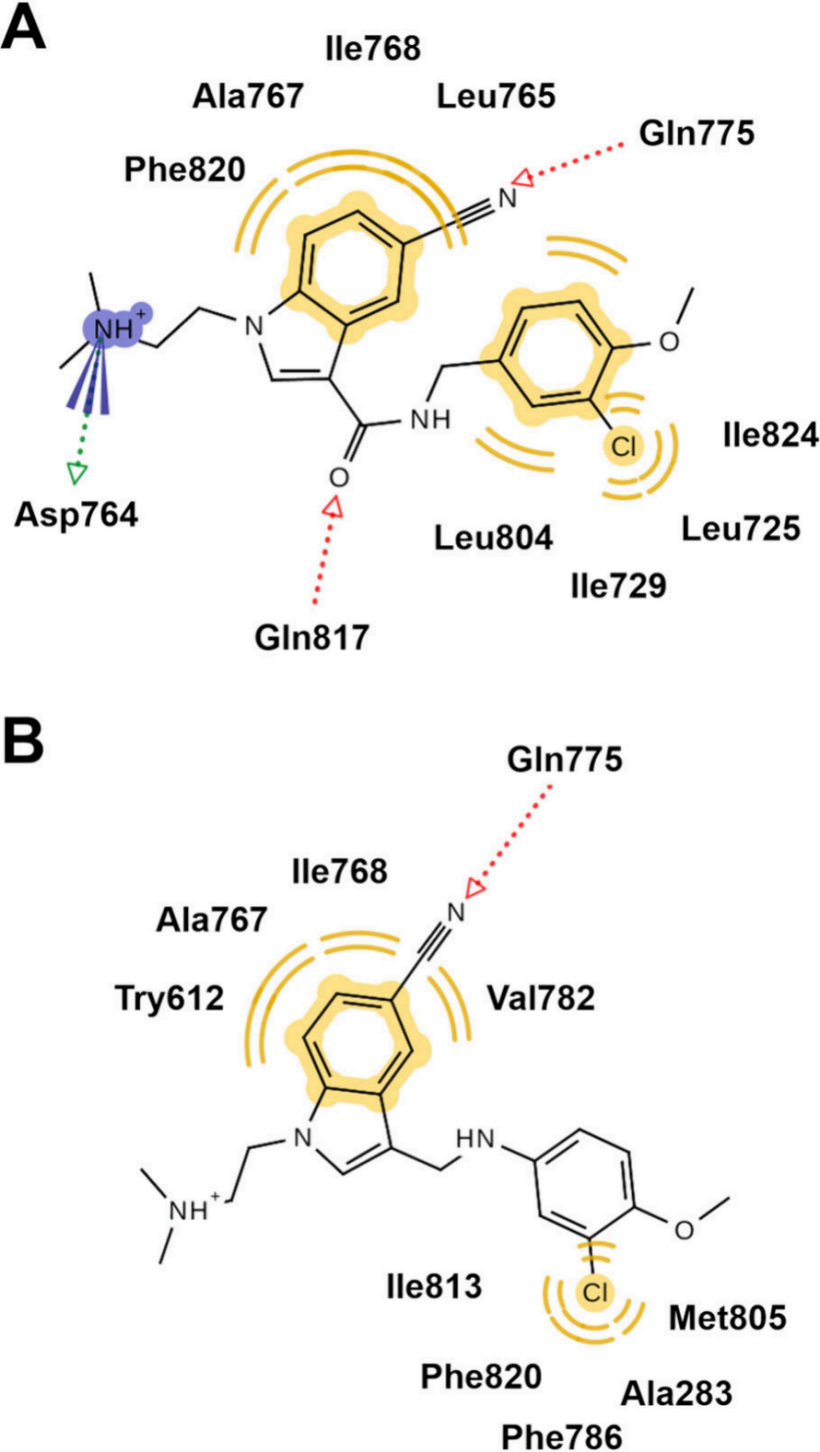
Two-dimensional representation of the pharmacophoric interactions of **14a** (A) and **5e** (B) as depicted by the LigandScout software based on the representative MD frame. Hydrophobic/aromatic interactions are highlighted by yellow curved lines. H-bonds are shown as colored dotted arrows (red = ligand is an H-bond acceptor; green = ligand is an H-bond donor); charged interactions are colored blue. For clarity, interactions with water molecules have been omitted.

**Table 2:** ChemPLP Dimensionless Docking Scores of **5e** and **14a** in the Two Main Poses Identified by Molecular Docking

**Compound**	**ChemPLP score** (nitrile group near Gln775)	**ChemPLP score** (nitrile group toward the solvent area)
**5e**	84.93	72.21
**14a**	85.17	N/A

Based on this computational study, we proposed new analogues of compound **14a** with structural modifications at those moieties deemed critical for the enzymatic binding (Scheme 3). Specifically, we removed the cyano group from the indole ring (compound **14c**). We also explored the contribution of the amide group on structural conformation, designing analogues with the nitrogen bearing an additional substituent (methyl group, compound **14d**) and with the benzene ring directly bonded to the nitrogen (compound **14b**).

These three analogues, **14b**–**d**, were then synthesized (Scheme 3) and tested for PDE5 enzymatic activity (Table 1). The synthetic route for **14c** and **14d** was identical to the one used for synthesizing **14a**. Particularly, for **14c**, we started with the commercially available indole-3-carcoxylic acid (**12c**), while for **14d**, we used the *N*-methylbenzylamine derivative. Analogue **14b** required a different synthetic approach, consisting of protecting the indole nitrogen of **11a** to form **11b** before performing the oxidation reaction to yield the carboxylic acid **12b**. Subsequently, the coupling reagent PyBrOP was employed to obtain the amide **13b**, which was deprotected and then alkylated to provide the final compound **14b**. NMR and HRMS data for compounds **14b**–**d** are reported in Supporting Information.

The enzymatic assay confirmed that removing the cyano group (compound **14c**) resulted in around a 1000-fold activity loss compared to **14a**, in agreement with previous findings on quinoline derivatives (Table 1).^13^ The *N*-phenyl amide analogue **14b** exhibited 100-fold reduced PDE5 activity compared to **14a**, supporting the binding mode described in our computational study, with the *N*-benzyl substituent ideally positioned in the enzymatic pocket. Moreover, **14c** produced about double the inhibition effect at 10 μM compared to compound **5e**. Such an outcome confirms that the carbonyl group in the amide series contributes to the compound-enzyme interaction by H-bonding to Gln817 as suggested by computational studies. Lastly, replacing the hydrogen of the amide with a methyl group (compound **14d**) induced a 2-log unit reduction of the enzymatic activity, further suggesting that the lack of steric hindrance and flexibility around the amide is critical for PDE5 binding and inhibition.

The cytotoxicity effect of compound **14a** was investigated to validate its suitability as a lead compound for further drug development. The cancer cell line MCF-7 was used in an MTT assay assessing cell metabolic activity (see Supporting Information for materials and methods). The compound showed a cytotoxic effect only at 100 μM, which is ∼ 6,000 times higher than its IC_50_ value, and after 72 h incubation, while it was safe at all other concentrations tested (Figure 5). Additionally, the cellular growth arrest observed in most conditions tested was attributed to the prensence of DMSO in the vehicle, as saline alone did not cause such an effect.

**Figure 5:**
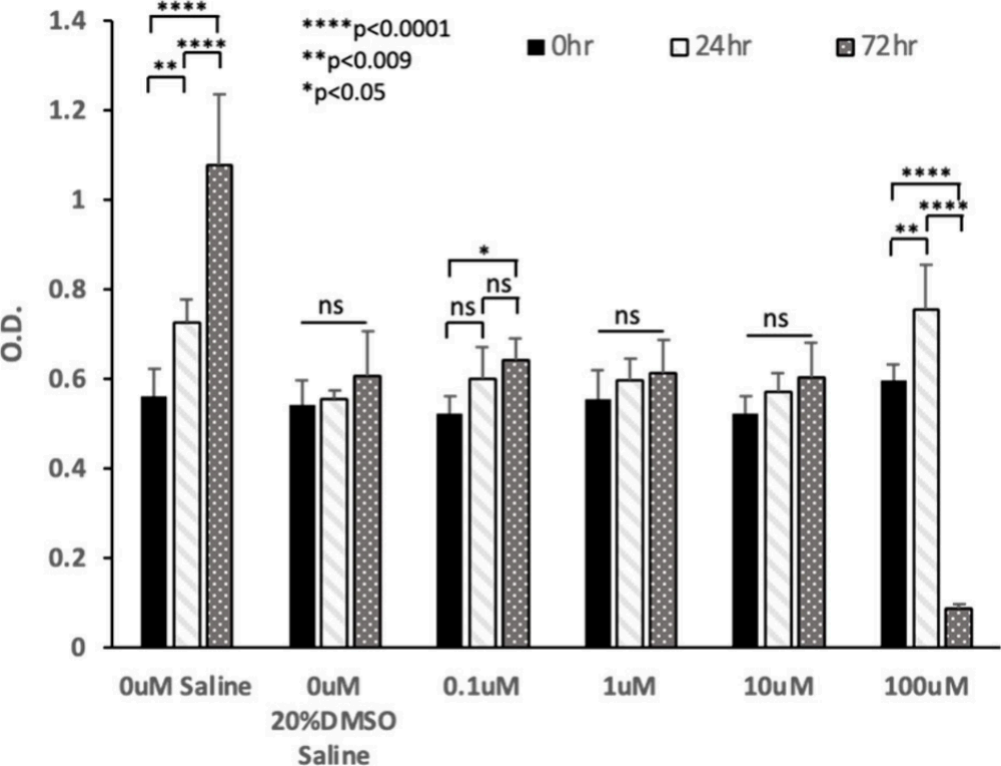
Cytotoxicity assay of compound **14a**. The compound (0.1–100 μM) was dissolved in 20% DMSO in saline; ns = nonsignificant.

Furthermore, we predicted the drug-like properties of compound **14a** compared to its amine counterpart **5e** and *N*-tosyl derivative **10**, using the SwissADME online tool (Table 3).^31^ The pharmacokinetic properties of compounds **14a** and **5e** were favorable, with moderate solubility in water, according to the ESOL solubility parameter. This data suggests a promising drug formulation potential, encouraging further research. The predicted lipophilicity value (logP < 4), together with its ability to cross the blood-brain barrier and a high GI absorbance, indicates good absorption and brain permeability, highlighting the potential of compound **14a** in future drug development. On the contrary, compound **10** was predicted to possess low GI absorption, poor aqueous solubility and blood-brain barrier (BBB) permeability.

**Table 3:** Estimated Drug-like Properties and Apparent Permeability (P_app_) of Compounds **5e**, **10**, and **14a**

Compound	Solubility (ESOL)	Consensus LogP	H-bond acceptors	H-bond donors	Lipinski violations	GI absorption	BBB Permeant	Bioavailability score	P_app_ ^a^ × 10^–6^ cm/s^–1^) (RSD)
**5e**	Moderately soluble	3.49	3	1	0	High	Yes	0.55	5.62 (24.80)
**10**	Poorly soluble	4.65	4	1	0	Low	No	0.55	n.d.
**14a**	Moderately soluble	3.19	4	1	0	High	Yes	0.55	2.83 (22.08)
**VER**	-	-	-	-	-	-	-	-	5.30 (16.01)
**CAF**	-	-	-	-	-	-	-	-	1.90 (10.09)

a P_app_ values for all compounds (tested at 50 μM) are the average of at least three independent experiments. n.d. = not detected. Verapamil (VER) and caffeine (CAF) were used as controls with higher and lower permeability, respectively, in the PAMPA assay.^29^

Lastly, we performed a PAMPA study of compounds **5e**, **10**, and **14a** using porcine brain lipids to measure the apparent permeability (P_app_) value according to the procedure described in Bicker *et al*. (Table 3).^29^ The P_app_ value of 2.0 × 10^–6^ *cm s* ^–1^ was identified as the cutoff to discriminate between BBB-permeable and nonpermeable compounds. Compounds with P_app_ higher than the cutoff value are considered BBB-permeable. Materials and methods for this study are reported in Supporting Information. Compounds **14a** and **5e** were found to have a P_app_ higher than the cutoff value, suggesting that they are likely to cross the BBB. Notably, the P_app_ data for **14a** and **5e** are in line with the SwissADME BBB permeability prediction. Finally, compound **10** was unable to cross the lipid layer in the PAMPA assay, confirming the predicted value.

In conclusion, this study investigated new indole-containing PDE5 inhibitors as potential treatments for various pathological conditions, including neurodegenerative diseases. Several structural modifications were performed on a scaffold derived from previously discovered PDE5 inhibitors (compounds **1** and **2**). Following enzymatic inhibition testing, compound **14a** emerged as the lead inhibitor, showing an IC_50_ of 16.11 nM. Structural modifications of the cyano and amide groups of **14a** were detrimental to its enzymatic binding mode, corroborating the importance of these two groups for the enzymatic activity. A cytotoxicity assay revealed that the compound was biologically safe up to 100 μM. Finally, PAMPA results confirmed its potential BBB permeability. These preliminary findings underscore compound **14a** as a promising candidate for further exploration of its pharmacokinetic properties and *in vivo* efficacy in animal models of AD.

## Supplementary Material







## Notes

Material and methods for chemistry, PDE5 enzymatic assay, cytotoxicity assay, and PAMPA; synthetic procedures of intermediates and final compounds; LC/MS analysis for final compounds; alternative docking pose of **5e**; MD simulations for the neutral form of compound **14a** (PDF)
^1^H NMR spectra for all final compounds (PDF)
^13^C NMR spectra for all final compounds (PDF)

## ○

International Flavors and Fragrances, 521 W 57th St, New York, NY

## □

Rutgers New Jersey Medical School, 185 S Orange Ave, Newark, NJ.

## ●

New York Institute of Technology, College of Osteopathic Medicine, Old Westbury, NY.
