# Supplementary material for: Discovery of Indole-Based PDE5 Inhibitors: Synthesis and Pharmacological Evaluation
Source: ACS Med Chem Lett. 2025 May 28;16(6):1058–65. doi: 10.1021/acsmedchemlett.5c00108 (PMC12169455; doi:10.1021/acsmedchemlett.5c00108)
Supplement: Supplementary file 1 [file ml5c00108_si_001.pdf]

Supporting Information

Discovery of Indole-Based PDE5 Inhibitors: Synthesis and Pharmacological Evaluation.

Shin-Young Park[1], Dang Pham[1], Param Shukla[1], Justin Edward[1], Reshmi John[1], Addison Li[1], Michael Hadjiargyrou[1], Mattia Mori[2], Elisa Zuccarello[3,4], Ottavio Arancio[3,4,5], and Jole Fiorito[1,4,*]

[1]Department of Biological and Chemical Sciences, New York Institute of Technology, Old Westbury, NY.
[2]Department of Biotechnology, Chemistry and Pharmacy, University of Siena, Italy.
[3]Taub Institute for Research on Alzheimer's Disease and the Aging Brain, Columbia University, New York, NY.
[4]Department of Medicine, Columbia University, New York, NY.
[5]Department of Pathology & Cell Biology, Columbia University, New York, NY.

Table of Contents **Pages**

1. Materials and Methods S1-S3
   1.1 Chemistry
   1.2 PDE5 Enzymatic Assay
   1.3 Cytotoxicity Assay
   1.4 PAMPA
2. Synthetic Procedures S4-S15
3. LC/MS Analysis S16
4. Figure S2 – Alternative docking pose of **5e** S17
5. Figure S3 – MD Simulations of the neutral form of compound **14a** S18

1. Materials and methods

1.1 Chemistry

All solvents and chemicals were purchased from Sigma-Aldrich, VWR, or Fisher Scientific. Solvents for liquid chromatography-mass spectrometry (LC/MS) analyses were Optima1 LC-MS grade (Fisher Scientific) or LiChrosolv1 LC/MS grade (Millipore). Purified recombinant human PDE5 (catalytic domain, amino acids 537–875, cat # 60050) was purchased from PBS Bioscience. Silica gel chromatography was performed using glass columns packed with silica gel (230−400 mesh, SiliCycle Inc.). ^{1}H and ^{13}C NMR spectra were recorded on an Agilent-NMR-vnmrs 400 (400 and 100MHz, respectively) spectrometer and were determined in chloroform-d and DMSO-d6, with tetramethylsilane (TMS) or solvent peaks as the internal reference. Chemical shifts (δ) are reported in ppm relative to the reference signal, coupling constant values are reported in Hertz (Hz), and signal multiplicity is indicated as follows: s = singlet, d = doublet, dd = doublet of doublets, t = triplet, dt = doublet of triplets, q = quartet, m = multiplet. Thin-layer chromatography (TLC) was performed on EMD pre-coated silica gel 60 F_{254} plates, and spots were visualized with UV light (254 nm). All air- or moisture-sensitive reactions were run under an argon atmosphere.

HRMS (TOF-ESI) was performed at Columbia University, Department of Chemistry. The purity of synthesized compounds was determined by LC/MS in scan mode. All synthesized compounds exhibited purities over 95%.

1.2 PDE5 Enzymatic Assay

Recombinant human PDE5 (BPS Bioscience, cat. no. 60050) was pre-incubated with the test compound in assay buffer (40 mM Tris HCl, pH 7.8, and 10 mM $MgCl_2$) for 15 minutes at room temperature. The maximum concentration of DMSO was 0.5% of the final reaction volume in all samples. The reaction was then initiated by adding 0.6 µM cGMP (Millipore) in assay buffer and incubated at 37 °C for 1 hour. The final reaction volume was 50 µl. Reactions were stopped by adding 50 µL of 0.1M HCl and neutralized with 2 µL of 2 M NaOH. The reaction mixture (40 µL) was analyzed via LC/MS to determine the amount of remaining cGMP. The following conditions were implemented in each experiment: blank, substrate control, positive control, and test compound (Table S1).

Table S1: Experimental conditions for PDE5 enzymatic activity assay.

Assay components	Blank (µL)	Substrate Control (SC) (µL)	Positive Control (PC) (µL)	Test Compound (µL)
cGMP (0.6 µM)	-	25	25	25
Assay buffer (40 mM TrisHCl, 10mM $MgCl_2$)	45	20	-	-
Test compound	-	-	-	5
5% DMSO in assay buffer	5	5	5	-
PDE5A1 (30pg/µl)	-	-	20	20
Total	50	50	50	50

LC/MS analysis was conducted using a Nexera-*i* LC-2040C Plus liquid chromatography system (Shimadzu), equipped with a 100 x 2.1mm C18 column (stainless steel, 3 µm silica, 110 Å pore, ACE Equivalence), and coupled to an LCMS-2020 single quadrupole mass spectrometer (Shimadzu). The components of the reaction mixture were separated using a ramp gradient of solvent A (0.1% formic acid in H_2O) and solvent B (acetonitrile) at a flow rate of 0.35 mL/min as follows: 0–2 minutes 0% B, 2–20 minutes 0–60% B, and 20–25 minutes 0% B.
The column oven and autosampler tray temperatures were maintained at 35 °C and 15 °C, respectively. The mass spectrometer operated in selected ion monitoring (SIM) mode with an m/z of 344.10 and a detection wavelength of 254 nm. The ionization mode was EPI; the DL temperature was set to 250 °C, the nebulizing gas flow to 1.5 L/min, and the heat block to 500 °C. For each sample, background subtraction was executed by using the blank sample as a background, and the chromatographic peak of cGMP was integrated to obtain the area.
Peak area data were analyzed using GraphPad Prism software to calculate the percentage of enzymatic activity. The peak area without the compound (PC = positive control) in each data set was defined as 100% activity. In the absence of PDE5 and the compound (SC = substrate control), the peak area value in each data set was defined as 0% activity. The percent activity with the compound was calculated using the following equation: % activity = (Peak Area of the compound – Peak Area of SC) / (Peak Area of PC – Peak Area of SC) × 100%. Figure S1 illustrates the concentration-percent activity curve for compound **14a**.
To validate the analytical method, a calibration curve using known concentrations of cGMP was generated using the LC/MS method described above to quantify the absolute amount of cGMP in each reaction sample. The linear range was from 1.875 nM to 0.3 µM. The limit of detection (LOD), defined as the lowest concentration producing a detectable peak above the background, was 0.003 µM. The limit of quantitation (LOQ), defined as the lowest amount of analyte in a sample that can be quantitatively determined with suitable precision and accuracy, was 0.009 µM ($R^2 > 0.95$). LOD and LOQ were calculated based on a calibration curve using the following equations: detection limit (DL)=3.3 σ/S and quantitation limit (QL)=10 σ/S, where σ is the standard deviation of the response, and S is the slope of the calibration curve.

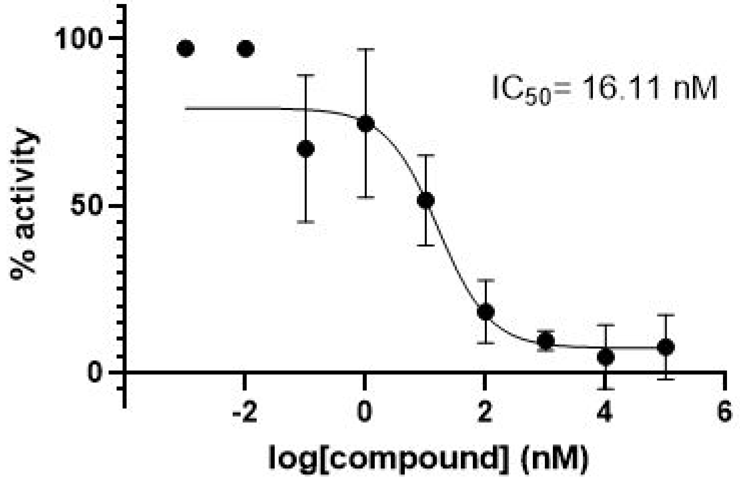

Figure S1. Concentration-percent activity curve for compound **14a**. Data is the average of three independent experiments.

1.3 Cytotoxicity Assay

MCF-7 mammary epithelial adenocarcinoma cells (ATCC, Manassas, VA, USA) were cultured in MEM (ATCC) supplemented with 10% fetal bovine serum and 100 I.U./mL penicillin and 50 μg/mL streptomycin (ATCC) in an environment of 5% CO_2 and 37 °C. For the cytotoxicity assay, cells were seeded in a 96-well tissue culture plate at 35 x 10^{-4} cells/well in quadruplicate/condition. After 24 hours, the drug was added to the cells at 0, 0.1, 1, 10, and 10 μM concentrations. Additionally, saline (0.9%) was added to the cells as a control. The cells were incubated with the drug for 24 and 72 hours, followed by the addition of MTT (3-(4, 5-dimethylthiazolyl-2)-2,5-diphenyltetrazolium bromide) reagent (ATCC) according to manufacturer's protocol. Briefly, at 24 and 72 hours, 10 mL MTT reagent was added to each well, and the plate was incubated for 2 hours at 37 °C, followed by the addition of 100 mL of detergent reagent and another 3 hours incubation at room temperature in the dark. The plate was then read at 570 nm in a spectrophotometer (Multiscan G0, Thermo Scientific) to obtain absorbance values.

1.4 PAMPA

A 2% solution of brain polar lipid extract (porcine) (cat. no. 141101P, Avanti Polar Lipid, Inc.) in *n*-dodecane was prepared, and 6 μL was added to the donor well membranes of a MultiScreen® filter plate (cat. no. MAIPNTR10, Millipore). Sample solutions (300 μL, 50 μM, 5% DMSO in PBS) of compounds **5e**, **14a**, **10**, verapamil, and caffeine were added to the donor wells. Then, 150 μL of blank solution (5% DMSO in PBS) was added to the acceptor wells (cat. no. MATRNPS50, Millipore). The donor wells were inserted into the acceptor wells and shaken at room temperature for 18 hours. Samples from the acceptor wells were collected and analyzed by HPLC (Nexera-*i* LC-2040C Plus, Shimadzu). Equilibrium solutions of compounds 5e, **14a**, 10, verapamil, and caffeine (33.3 μM, 5% DMSO in PBS) were also prepared and analyzed by HPLC. The assay was performed in triplicate.

A 100 x 2.1 mm C18 column (stainless steel, 3 μm silica, 110 Å pore, ACE Equivalence) was used for the HPLC analysis. Sample solutions were separated using a ramp gradient of solvent A (0.1% formic acid in H_2O) and solvent B (acetonitrile) at a flow rate of 0.4 mL/min, as follows: 0–1 minute: 10% B, 1–10 minutes: 10–100% B, 10–12 minutes: 100% B, and 12-15 minutes: 10% B. The column oven and autosampler tray temperatures were maintained at 28 ℃ and 15 ℃, respectively. Peak area data of sample and equilibrium solutions were used to calculate the apparent permeability (P_{app}) using the following equation:

$$P_{app} = C \times - \ln\left(1 - \frac{AUCa}{AUCe}\right) cm/s$$

where $C = \frac{V_d \times V_a}{(V_d + V_a) \times Area \times time} cm/s$, AUCa is the area under the curve of the acceptor solution minus blank, and AUCe is the area under the curve of the equilibrium solution minus blank. In this protocol, the donor volume (V_d) is 0.3 cm^3, acceptor volume (V_a) is 0.15 cm^3, membrane area (Area) is 0.26 cm^2, and time is 64,800 s.

2. Synthetic Procedures

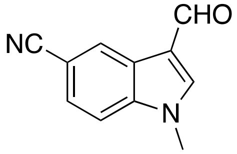

3-formyl-1-methyl-1*H*-indole-5-carbonitrile (4a). 1*H*-indole-5-carbonitrile (2.0 g, 11.75 mmol) was dissolved in THF (25 mL). NaH (564 mg, 23 mmol) was added portion-wise at 0°C. The reaction mixture was stirred for 1 h under argon and at rt, and CH_3I (1.43 mL, 23 mmol) was added dropwise to the reaction. The reaction was stopped after stirring for 3 h. The product was filtered over a plug of celite and washed with dichloromethane, then extracted with NH_4Cl (2x50mL) and water (1x50mL). The organic layer was evaporated under vacuum to yield the desired product quantitatively. LC/MS (ESI) m/z 226.05 $[M+CH_3CN+H]^+$.

General synthetic procedure for the synthesis of *N*-alkyl 3-formyl-5-cyanoindoles (4b-g). 3-formyl-1*H*-indole-5-carbonitrile was dissolved in DMF, and Cs_2CO_3 (1.5 equiv) was added. The corresponding alkyl halide (1.5 equiv) was added, and the reaction was stirred at 80°C for 3-20 h. After the starting material was consumed (monitored by TLC analysis), AcOEt was added, and the mixture was washed with H_2O (4 times). The organic layer was dried over Na_2SO_4, filtered, and evaporated under reduced pressure to afford the desired product, which was used without further purification unless specified.

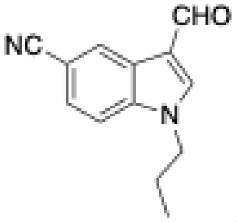

3-formyl-1-propyl-1H-indole-5-carbonitrile (4b). TLC in hexane/AcOEt (1:1). Yield: 100%. LC/MS (ESI) m/z 254.20 $[M+CH_3CN+H]^+$.

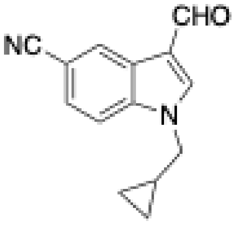

1-(cyclopropylmethyl)-3-formyl-1*H*-indole-5-carbonitrile (4c). TLC in hexane/AcOEt. (1:1). Yield: 67.5%. LC/MS (ESI) m/z 266.20 $[M+CH_3CN+H]^+$.

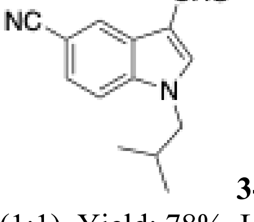

3-formyl-1-isobutyl-1*H*-indole-5-carbonitrile (4d). TLC in hexane/AcOEt. (1:1). Yield: 78%. LC/MS (ESI) m/z 268.20 $[M+CH_3CN+H]^+$.

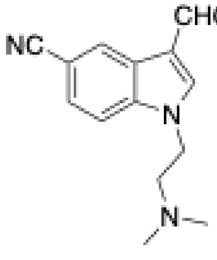

1-(2-(dimethylamino)ethyl)-3-formyl-1*H*-indole-5-carbonitrile (4e). TLC in DMC/MeOH (9.5:0.5). Yield: 67.5%. LC/MS (ESI) m/z 283.00 [M+CH_3CN+H]$^+$.

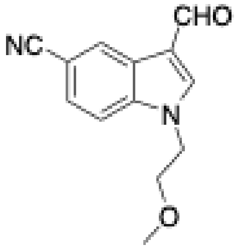

3-formyl-1-(2-methoxyethyl)-1*H*-indole-5-carbonitrile (4f). TLC in DMC/MeOH (9.5:0.5). Yield: 90.6%. LC/MS (ESI) m/z 270.00 [M+ CH_3CN+H]$^+$.

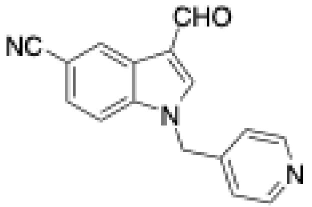

3**-formyl-1-(pyridin-4-ylmethyl)-1*H*-indole-5-carbonitrile (4g).** Both TLC and column chromatography in AcOEt. Yield: 67.5%. LC/MS (ESI) m/z 262.05 [M+H]$^+$.

General procedure for the synthesis of compounds 5a-k. *N*-substituted 3-formyl-1*H*-indole-5-carbonitrile (1 equiv) and appropriate amine (1.2 equiv) were dissolved in methanol and stirred at room temperature under argon for 18-24 h. The reaction was placed in an ice bath and cooled down to 0℃ and $NaBH_4$ (2 equiv) was added. The mixture was stirred at room temperature for 1-4 h. The solvent was evaporated under vacuum, and the residue was dissolved in EtOAc and washed with H_2O (3x). The organic layer was evaporated under vacuum and purified by column chromatography.

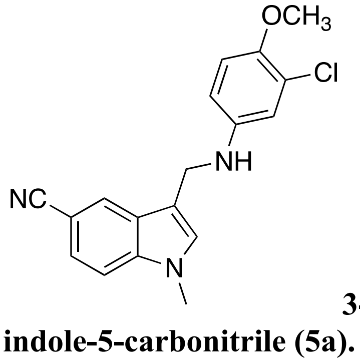

3-(((3-chloro-4-methoxyphenyl)amino)methyl)-1-methyl-1*H*-indole-5-carbonitrile (5a). Column chromatography in hexane/AcOEt 3:2. Yield: 60% (off-white solid). ^{1}H NMR (400 MHz, DMSO-d$_6$) δ 8.202 (s, 1H), 7.600 (d, J = 8.8 Hz, 1H), 7.503-

7.483 (m, 2H), 6.886 (d, *J* = 9.2 Hz, 1H), 6.721 (d, *J* = 2.4 Hz, 1H), 6.590 (dd, *J* = 2.8, 9.2 Hz, 1H), 5.902 (t, *J* = 5.6 Hz, 1H, $CH_2\underline{NH}$), 4.349 (d, *J* = 6 Hz, 2H, $\underline{CH_2}NH$), 3.796 (s, 3H), 3.683 (s, 3H). ^{13}C NMR (101 MHz, DMSO-d_6) δ 145.58, 143.79, 138.25, 130.46, 126.61, 124.85, 123.78, 121.71, 120.71, 114.58, 113.66, 113.40, 111.60, 111.08, 100.52, 56.56, 38.45, 32.60. HRMS (ESI), m/z $[M+Na]^+$, calcd for $C_{18}H_{16}ClN_3ONa$: 348.0880, found 348.0880.

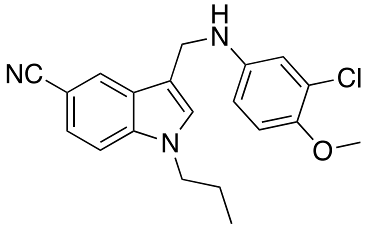

3-(((3-chloro-4-methoxyphenyl)amino)methyl)-1-propyl-1*H*-indole-5-carbonitrile (5b). Column chromatography in 1% MeOH/DCM. Yield: 81% (colorless oil). ^{1}H NMR (400 MHz, DMSO-d_6) δ 8.207 (d, 1H, *J* = 0.8 Hz), 7.647 (d, 1H, *J* = 8.8 Hz), 7.554 (s, 1H), 7.465 (dd, 1H, J = 1.6, 8.8 Hz), 6.883 (d, 1H, *J* = 9.2 Hz), 6.729 (d, 1H, *J* = 2.8 Hz), 6.601 (dd, 1H, *J* = 2.8, 8.8 Hz), 5.892 (t, 1H, *J* = 5.6 Hz, $\underline{NH}CH_2$), 4.353 (d, 2H, *J* = 5.2 Hz, $NH\underline{CH_2}$), 4.147 (t, 2H, *J* = 7.2 Hz), 3.683 (s, 3H), 1.726 (sextet, 2H, *J* = 7.2 Hz), 0.796 (t, 3H, *J* = 7.6 Hz). ^{13}C NMR (101 MHz, DMSO-d_6) δ 145.59, 143.83, 137.79, 129.69, 126.67, 124.99, 123.72, 121.70, 120.72, 114.58, 113.66, 113.36, 111.76, 111.20, 100.47, 56.57, 47.06, 38.63, 23.13, 11.02. HRMS (ESI), m/z $[M+Na]^+$, calcd for $C_{20}H_{20}ClN_3ONa$: 376.1193, found 376.1181.

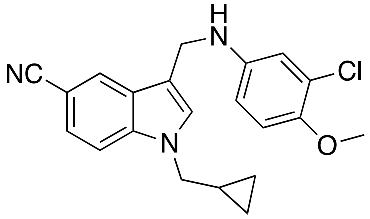

3-(((3-chloro-4-methoxyphenyl)amino)methyl)-1-(cyclopropylmethyl)-1*H*-indole-5-carbonitrile (5c). Column chromatography in Hexane/AcOEt 8:2. Yield: 65.0% (off-white solid). ^{1}H NMR (400 MHz, DMSO-d_6) δ 8.207 (s, 1H), 7.685 (d, 1H, *J* = 8.4 Hz), 7.620 (s, 1H), 7.469 (dd, 1H, *J* = 1.6, 8.8 Hz), 6.891 (d, 1H, *J* = 8.8 Hz), 6.744 (d, 1H, *J* = 2.8 Hz), 6.611 (dd, 1H, *J* = 2.4, 8.8 Hz), 5.894 (t, 1H, *J* = 6.0 Hz, $\underline{NH}CH_2$), 4.359 (d, 2H, *J* = 6.0 Hz, $NH\underline{CH_2}$), 4.067 (d, 2H, *J* = 7.2 Hz), 3.689 (s, 3H), 1.194 (septet, 1H, *J* = 2.8 Hz), 0.517-0.472 (m, 2H), 0.390-0.352 (m, 2H). ^{13}C NMR (101 MHz, DMSO-d_6) δ 145.49, 143.76, 137.64, 129.42, 126.62, 124.83, 123.63, 121.60, 120.62, 114.48, 113.54, 113.30, 111.63, 111.18, 100.39, 56.46, 49.59, 38.54, 11.39, 3.55. HRMS (ESI), m/z $[M+Na]^+$, calcd for $C_{21}H_{20}ClN_3ONa$: 388.1193, found 388.1199.

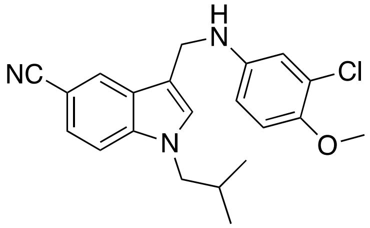

3-(((3-chloro-4-methoxyphenyl)amino)methyl)-1-isobutyl-1*H*-indole-5-carbonitrile (5d). Column chromatography in DCM. Yield: 61.5% (off-white solid). ^{1}H NMR (400 MHz, DMSO-d_6) δ 8.211 (d, 1H, *J* = 1.2 Hz), 7.650 (d, 1H, *J* = 8.4 Hz), 7.523 (s,

1H), 7.458 (dd, 1H, *J* = 1.6, 8.8 Hz), 6.875 (d, 1H, *J* = 9.2 Hz), 6.720 (d, 1H, *J* = 2.8 Hz), 6.596 (dd, 1H, *J* = 2.8, 9.2 Hz), 5.908 (t, 1H, *J* = 6.0 Hz, <u>NH</u>CH_2), 4.359 (d, 2H, *J* = 6.0 Hz, NH<u>CH_2</u>), 3.993 (d, 2H, *J* = 7.2 Hz), 3.678 (s, 3H), 2.056 (septet, 1H, *J* = 6.4 Hz), 0.815 (d, 6H, *J* = 6.4 Hz). ^{13}C NMR (101 MHz, DMSO-d_6) δ 145.57, 143.79, 138.09, 130.29, 130.10, 126.59, 125.01, 121.65, 120.75, 114.52, 113.67, 113.61, 113.25, 111.87, 100.46, 56.63, 52.73, 38.63, 29.23, 19.77. HRMS (ESI), m/z $[M+Na]^+$, calcd for $C_{21}H_{22}ClN_3ONa$: 390.1349, found 390.1349.

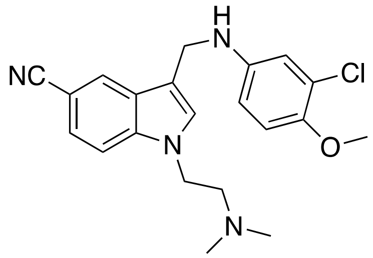

3-(((3-chloro-4-methoxyphenyl)amino)methyl)-1-(2-(dimethylamino)ethyl)-1*H*-indole-5-carbonitrile (5e). Column chromatography in 4% MeOH/DCM. Yield: 38.7% (off-white solid). ^{1}H NMR (400 MHz, DMSO-d_6) δ 8.192 (d, 1H, *J* = 1.2 Hz), 7.651 (d, 1H, *J* = 8.8 Hz), 7.563 (s, 1H), 7.462 (dd, 1H, *J* = 8.0, 1.2 Hz), 6.884 (d, 1H, *J* = 9.2 Hz), 6.728 (d, 1H, *J* = 2.8 Hz), 6.600 (dd, 1H, *J* = 2.8, 8.8 Hz), 5.889 (t, 1H, *J* = 6.0 Hz, <u>NH</u>CH_2), 4.345 (d, 2H, *J* = 6.0 Hz, NH<u>CH_2</u>), 4.262 (t, 2H, *J* = 6.4 Hz), 3.685 (s, 3H), 2.574 (t, 2H, *J* = 6.4 Hz), 2.146 (s, 6H). ^{13}C NMR (101 MHz, DMSO-d_6) δ 145.59, 143.86, 137.89, 129.85, 126.66, 124.91, 123.73, 121.72, 120.74, 114.59, 113.65, 113.35, 111.75, 111.18, 100.46. 58.64, 56.59, 45.24, 43.66, 38.66. HRMS (ESI), m/z $[M+Na]^+$, calcd for $C_{21}H_{23}ClN_4ONa$: 405.1458, found 405.1473.

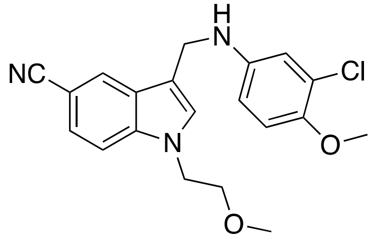

3-(((3-chloro-4-methoxyphenyl)amino)methyl)-1-(2-methoxyethyl)-1*H*-indole-5-carbonitrile (5f). Column chromatography in Hexane/AcOEt 6:4. Yield: 73.7% (off-white solid). ^{1}H NMR (400 MHz, DMSO-d_6) δ 8.199 (d, 1H, *J* = 0.8 Hz), 7.652 (d, 1H, *J* = 8.4 Hz), 7.527 (s, 1H), 7.470 (dd, 1H, *J* = 1.2, 8.4 Hz), 6.888 (d, 1H, *J* = 8.8 Hz), 6.736 (d, 1H, *J* = 2.8 Hz), 6.606 (dd, 1H, *J* = 2.4, 8.8 Hz), 5. 896 (t, 1H, *J* = 5.6 Hz, <u>NH</u>CH_2), 4.358-4.337 (m, 4H, NH<u>CH_2</u> and <u>CH_2</u>OCH_3), 3.687 (s, 3H), 3.620 (t, 2H, *J* = 4.8 Hz), 3.318 (s, 3H). ^{13}C NMR (101 MHz, DMSO-d_6) δ 145.64, 143.91, 138.08, 129.92, 126.75, 124.93, 123.79, 121.76, 120.76, 114.63, 113.68, 113.52, 111.77, 111.42, 100.60, 71.06, 58.13, 56.66, 45.54, 38.65. HRMS (ESI), m/z $[M+Na]^+$, calcd for $C_{20}H_{20}ClN_3O_2Na$: 392.1142, found 392.1133.

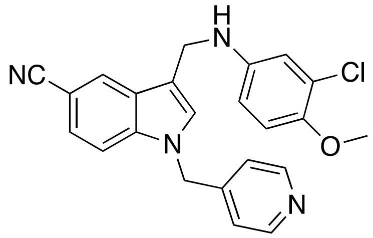

3-(((3-chloro-4-methoxyphenyl)amino)methyl)-1-(pyridin-4-ylmethyl)-1*H*-indole-5-carbonitrile (5g). Column chromatography in 1% MeOH/DCM. Yield: 40.0% (white solid). ^{1}H NMR (400 MHz, DMSO-d$_6$) δ 8.459 (dd, 2H, J = 1.6, 4.8 Hz), 8.264 (d, 1H, J = 0.8 Hz), 7.671 (s, 1H,), 7.595 (d, 1H, J = 8.8 Hz), 7.468 (dd, 1H, J = 1.2, 8.0 Hz), 7.029 (d, 2H, J = 5.6 Hz),6.889 (d, IH, J = 8.4 Hz), 6.727 (d, 1H, J = 2.8 Hz), 6.602 (dd, 1H, J = 2.4, 8.4 Hz), 5.958 (t, 1H, J = 6.4 Hz, CH$_2$<u>NH</u>), 5.533 (s, 2H, N<u>CH$_2$</u>Pyr), 4.399 (d, 2H, J = 5.2 Hz, <u>CH$_2$</u>NH), 3.690 (s, 3H, OCH$_3$). ^{13}C NMR (101 MHz, DMSO-d$_6$) δ 149.86, 149.84, 146.63, 145.66, 143.74, 137.92, 130.17, 130.11, 126.95, 125.20, 124.31, 121.70, 121.67, 120.52, 114.57, 114.38, 113.72, 111.95, 111.40, 101.19, 56.63, 47.91, 38.63. HRMS (ESI), m/z [M+Na]$^+$, calcd for $C_{23}H_{19}ClN_4ONa$: 425.1145, found 425.1144.

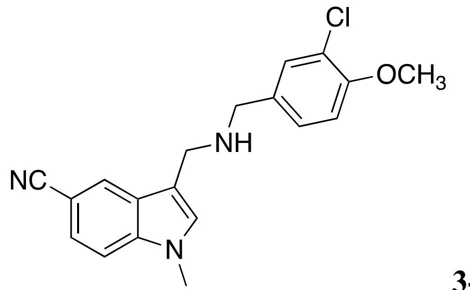

3-(((3-chloro-4-methoxybenzyl)amino)methyl)-1-methyl-1*H*-indole-5-carbonitrile (5h). Column chromatography in 2% MeOH/DCM. Yield: 72% (white solid). ^{1}H NMR (400 MHz, DMSO-d$_6$) δ 8.137 (1H, d, J = 0.8 Hz), 7.587 (1H, d, J = 8.8 Hz), 7.479 (1H, dd, J = 1.2, 8.4 Hz), 7.417 (1H, s), 7.400 (1H, d, J = 1.6 Hz), 7.246 (1H, dd, J = 1.6, 8.4 Hz), 7.071 (1H, d, J = 8.4 Hz), 3.827 (s, 3H, O<u>CH$_3$</u>), 3.819 (s, 2H), 3.801 (s, 3H, N<u>CH$_3$</u>), 3.647 (s, 2H). ^{13}C NMR (101 MHz, DMSO-d$_6$) δ 153.18, 138.29, 134.07, 130.55, 129.32, 127.81, 127.00, 124.91, 123.74, 120.82, 120.66, 114.18, 112.42, 111.02, 100.39, 56.04, 50.96, 42.86, 32.58. HRMS (ESI), m/z [M+H]$^+$, calcd for $C_{19}H_{18}ClN_3O$: 340.1217, found 340.1213.

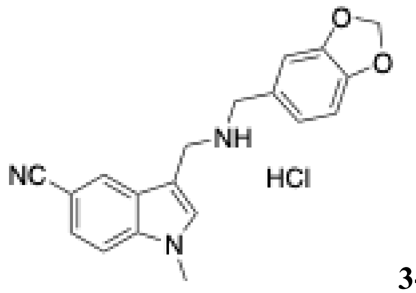

3-(((benzo[*d*][1,3]dioxol-5-ylmethyl)amino)methyl)-1-methyl-1*H*-indole-5-carbonitrile hydrochloride (5i). Column chromatography in 4% MeOH/DCM yielded the desired product as a colorless oil. The product was treated with HCl Et_2O 2.0 M to obtain its corresponding hydrochloride salt. Yield: 78% (white solid). ^{1}H NMR (400 MHz, DMSO-d$_6$) δ 9.312 (s, 2H), 8.349 (s, 1H), 7.744 (s, 1H), 7.690 (d, J = 8.4 Hz, 1H), 7.573 (d, J =

8.8 Hz, 1H), 7.148 (s, 1H),7.003-6.945 (m, 2H), 6.049 (s, 2H, O<u>CH</u>$_2$O), 4.318 (s, 2H), 4.083 (s, 2H), 3.872 (s, 3H). ^{13}C NMR (101 MHz, DMSO-d$_6$) δ 147.56, 147.28, 138.01, 134.00, 126.79, 125.18, 124.23, 123.88, 120.51, 111.47, 110.11, 108.23, 101.33, 101.27., 49.44, 40.45, 32.92. HRMS (ESI), m/z [M]$^+$, calcd for $C_{19}H_{18}N_3O_2$: 320.1399, found 320.1396.

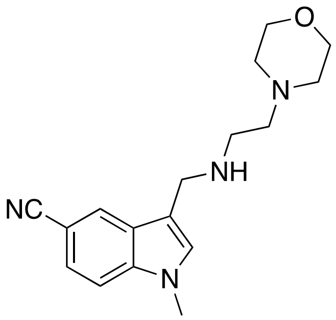

1-methyl-3-(((2-morpholinoethyl)amino)methyl)-1*H*-indole-5-carbonitrile (5j). Column chromatography in 5% MeOH/DCM. Yield: 60.2% (off-white solid). ^{1}H NMR (400 MHz, DMSO-d$_6$) δ 8.275 (s, 1H), 7.636 (d, 1H, J = 8.0 Hz), 7.557 (s, 1H), 7.525 (dd, 1H, J = 1.6, 8.8 Hz), 4.073 (s, 2H, CH$_2$NH), 3.831 (s, 3H, NCH$_3$), 3.541 (t, 4H, J = 4.4 Hz, morpholine), 2.791 (t, 2H, J = 6.4 Hz), 2.450 (t, 2H, J = 6.4 Hz), 2.325 (s, 4H, morpholine). ^{13}C NMR (101 MHz, DMSO-d$_6$) δ 138.17, 132.08, 126.91, 124.97, 123.98, 120.64, 111.25, 100.84, 66.09, 55.84, 53.20, 43.81, 42.30, 32.71. HRMS (ESI), m/z [M+H]$^+$, calcd for $C_{17}H_{22}N_4O$: 299.1872, found 299.1867.

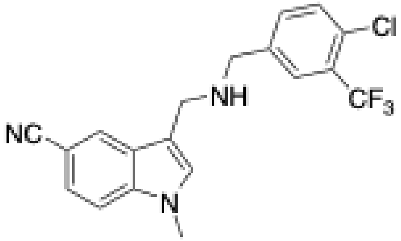

3-(((4-chloro-3-(trifluoromethyl)benzyl)amino)methyl)-1-methyl-1*H*-indole-5-carbonitrile (5k). Column chromatography in hexane/AcOEt 3:7. Yield: 71.9% (white solid). ^{1}H NMR (400 MHz, DMSO-d$_6$) δ 8.144 (d, 1H, J = 1.2 Hz), 7.803 (s, 1H), 7.624 (s, 2H), 7.580 (d, 1H, J = 8.4 Hz), 7.476 (dd, 1H, J = 1.2, 9.2 Hz), 7.409 (s, 1H), 3.845 (s, 2H), 3.791 (s, 3H), 3.770 (s, 2H). ^{13}C NMR (101 MHz, DMSO-d$_6$) δ 141.35, 138.17, 133.35, 131.03, 130.43, 128.20, 126.82, 126.22, 125.92, 124.74, 123.60, 122.88 (q, J_{CF} = 274.32 Hz), 120.62, 114.13, 110.87, 100.29, 50.75, 42.98, 32.40. HRMS (ESI), m/z [M+Na]$^+$, calcd for $C_{19}H_{15}ClF_3N_3Na$: 400.0804, found 400.0802.

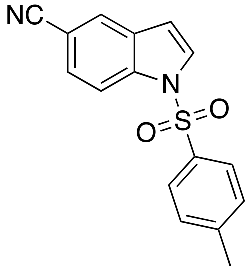

1-tosyl-1*H*-indole-5-carbonitrile (7). 1*H*-indole-5-carbonitrile (0.5 g, 3.5 mmol), NaOH (0.252 g, 6.3 mmol), and TEBA (0.0797 g, 0.35 mmol) were dissolved in DCM (1.5 mL), and then tosyl chloride (0.804g, 4.2 mmol) was added. The reaction was stirred at

room temperature for 24 h. The reaction mixture was partitioned between H_2O and DCM, and the organic layer was separated and washed with brine (3x), dried over $NaSO_4$, filtered, evaporated under vacuum, and used without further purification. Yield: 80% (white solid). LC/MS (ESI) m/z 338.00 $[M+H+ACN]^+$.

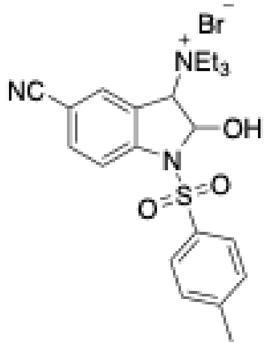

5-cyano-*N*,*N*,*N*-triethyl-2-hydroxy-1-tosylindolin-3-aminium bromide (8). NBS (1.1 equiv) and H_2O (10 equiv) were added to a solution of **7** in acetone. The reaction was stirred at room temperature for 4.5 h. Et_3N (1.1 equiv) was added to the reaction mixture, and the reaction was stirred for 2 h at room temperature. The solvent was evaporated, and the residue was purified by column chromatography in 10% MeOH/DCM. Yield: 84% (white solid). LC/MS (ESI) m/z 415.10 $[M+H]^+$.

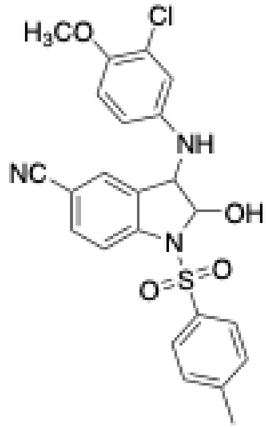

3-((3-chloro-4-methoxyphenyl)amino)-2-hydroxy-1-tosylindoline-5-carbonitrile (9). 3-chloro-4-methoxyaniline (1.1 equiv) and Et_3N (2 equiv) were added to a solution of **8** (700 mg, 1.42 mmol) in AcOEt, and the reaction was refluxed for 1.5 h. The reaction was cooled down and filtered to remove the solid. The filtrate was evaporated, and the residue was purified by column chromatography in Hexane/AcOEt 8:2. Yield: 35% (pink solid). LC/MS (ESI) m/z 468.00 $[M–H]^-$.

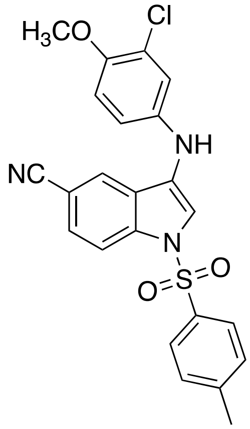

3-((3-chloro-4-methoxyphenyl)amino)-1-tosyl-1*H*-indole-5-carbonitrile (10). Compound **9** (92 mg, 0.196 mmol) was dissolved in AcOEt (2.0 mL), and BF_3 Et_2O (120.8 µl, 0.98 mmol) was added. The reaction was stirred at 50 °C overnight. The resulting precipitate was collected by filtration, washed with AcOEt, and dried under vacuum to give the desired product **10.** Yield: 41% (pink solid). ^{1}H NMR (400 MHz, DMSO-d_6) δ 8.306 (d, 1H, J = 1.2 Hz), 8.206 (s, 1H), 8.130 (d, 1H, 8.4 Hz), 7.891 (d, 2H, J = 8.4 Hz), 7.803 (dd, 1H, J = 1.2, 8.4 Hz), 7.529 (s, 1H), 7.370 (d, 2H, J = 8.4 Hz), 7.155 (d, 1H, J = 2.0 Hz), 7.126-1.120 (m, 2H), 3.819 (s, 3H, O<u>CH</u>$_3$), 2.306 (s, 3H, <u>CH</u>$_3$Ph). ^{13}C NMR (101 MHz, DMSO-d_6) δ 148.37, 145.77, 137.34, 135.20, 133.24, 130.27, 128.63, 126.97, 126.83, 126.17, 124.51, 121,57, 119.06, 117.89, 115.51, 114.83, 114.00, 108.63, 105.5. HRMS (ESI), m/z 452.15 [M+H]$^+$, calcd for $C_{23}H_{18}ClN_3O_3S$: 452.0836, found 452.0815.

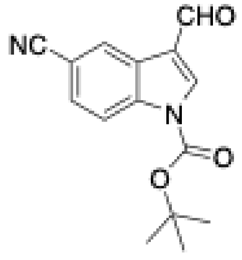

***tert*-butyl 5-cyano-3-formyl-1*H*-indole-1-carboxylate (11b)**. DMAP (0.1 equiv) and Boc_2O (1.1 equiv) were added to a suspension of 3-formyl-1*H*-indole-5-carbonitrile (11a) in DCM. The reaction mixture was stirred at room temperature for 24 h. The resulting solid was collected by vacuum filtration. Yield: 87% (white solid).

General procedure for the synthesis of compounds 12a-b. $NaOCl_2$ (4 equiv) was added to a suspension of 3-formyl-1*H*-indole, 2-methyl-2-butene (5 equiv), $NaHPO_4$ (1.5 equiv) in a solution of *t*-butanol/H_2O/MeOH (4:1:4). The reaction was stirred at rt for 1-4 days. The reaction was acidified by adding HCl (1.0 M), and the resulting precipitate was collected by vacuum filtration and used without further purification.

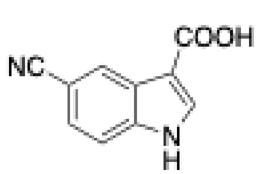

5-cyano-1*H*-indole-3-carboxylic acid (12a). Yield: 100% (white solid). LCMS (ESI) m/z 184.90 [M-H]$^+$.

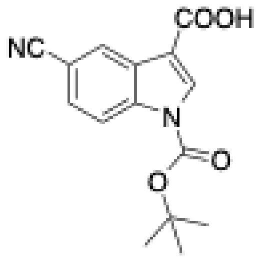 **1-(*tert*-butoxycarbonyl)-5-cyano-1H-indole-3-carboxylic acid (12b)**. Yield: 63% (white solid). LCMS (ESI) m/z 285.00 [M-H]$^+$.

General procedure for the synthesis of compounds 13a, 13d, and 13e. EDC (2 equiv) was added to a solution of carboxylic acid (1 equiv), amine (1.5 equiv), DMAP (1.1 equiv), and DIPEA (4 equiv) in DCM. The reaction mixture was stirred rt for 3 h to 4 days.

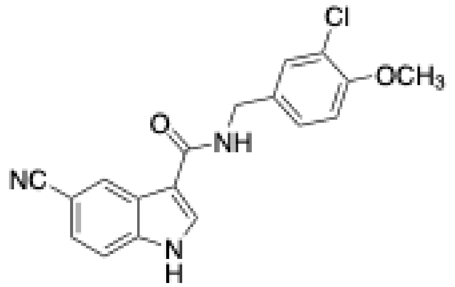 ***N*-(3-chloro-4-methoxybenzyl)-5-cyano-1*H*-indole-3-carboxamide (13a)**. The product was collected by recrystallization in methanol. Yield: 51% (white solid). LC/MS (ESI) m/z 340.10 [M+H]$^+$.

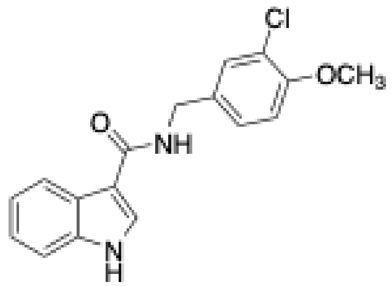 ***N*-(3-chloro-4-methoxybenzyl)-1*H*-indole-3-carboxamide (13d)**. The resulting precipitate was collected by vacuum filtration. Yield 86% (white solid). LC/MS (ESI) m/z 315.00 [M+H]$^+$.

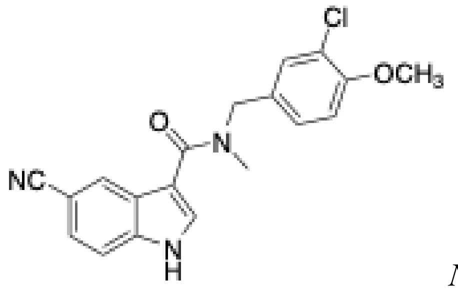 ***N*-(3-chloro-4-methoxybenzyl)-5-cyano-*N*-methyl-1*H*-indole-3-carboxamide (13e)**. The reaction mixture was extracted with HCl (0.1 M) (2 times) and brine (1 time), The organic layer was dried over Na_2SO_4 and evaporated. Column

chromatography in 3% MeOH/DCM. Yield 31.6% (white solid). LC/MS (ESI) m/z 354.05 $[M+H]^+$.

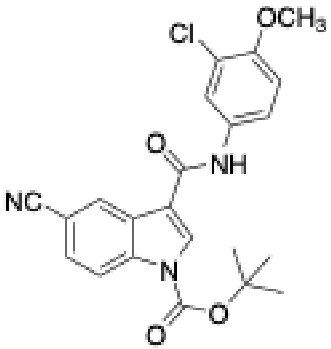

***tert*-butyl 3-((3-chloro-4-methoxyphenyl)carbamoyl)-5-cyano-1*H*-indole-1-carboxylate (13b).** DIPEA (4 equiv), 3-chloro-4-methoxyaniline (1 equiv), PyBroP (1.5 equiv) were added to a solution of **12b** in DCM. The reaction was stirred at 40°C for 24 h. The resulting precipitate was collected by vacuum filtration. Yield: 51% (white solid). LC/MS (ESI) m/z 426.10 $[M+H]^+$.

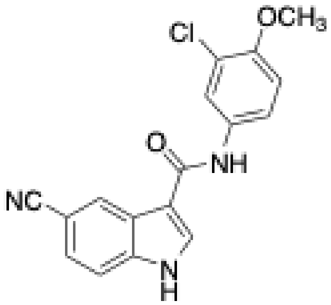

***N*-(3-chloro-4-methoxyphenyl)-5-cyano-1*H*-indole-3-carboxamide (13c)**. Compound **13b** (200 mg) was dissolved in DCM (4 mL) and TFA (2 mL) and stirred at room temperature for 2.5 h. Solvent was evaporated, and the residue was dissolved in AcOEt and washed with H_2O (2 times) and brine (1 time). The organic layer was dried over Na_2SO_4 and evaporated to yield **13c** (90.2%) as a white solid. LC/MS (ESI) m/z 326.05 $[M+H]^+$.

General procedure for the synthesis of compounds 14a-d. A mixture of amide and Cs_2CO_3 (2.5 equiv) was stirred for 30 minutes at 80 °C. 2-chloro-*N*,*N*-dimethylethan-1-amine hydrochloride (1.5 equiv) was added to the mixture and stirred overnight at 80 °C. The reaction was partitioned between AcOEt and H_2O and washed with H_2O (3x). The organic layer was evaporated under vacuum and purified by column chromatography.

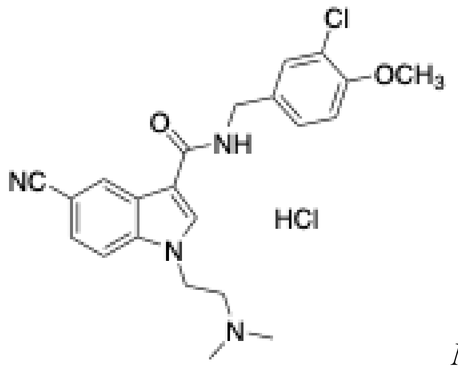

***N*-(3-chloro-4-methoxybenzyl)-5-cyano-1-(2-(dimethylamino)ethyl)-1*H*-indole-3-carboxamide hydrochloride (14a)**. Column chromatography: 5% MeOH in DCM. Yield: 48% (white solid). The final product was converted into its hydrochloride form using 2N HCl in ether. ^{1}H NMR (400 MHz, DMSO-d$_6$) δ 10.840 (s, 1H, <u>NH</u>$^{+}$(CH$_3$)$_2$), 8.746 (t, 1H, J = 5.2 Hz, CO<u>NH</u>), 8. 572 (d, 1H, J = 0.8 Hz), 8.363 (s, 1H), 7.936 (d, 1H, J = 8.4 Hz), 7.672 (dd, 1H, J = 1.6, 8.8 Hz), 7.401 (d, 1H, J = 2.0 Hz), 7.295 (dd, 1H, J = 2.0, 8.4 Hz), 7.109 (d, 1H, J = 8.0 Hz), 4.755 (t, 2H, J = 6.8 Hz), 4.417 (d, 2H, J = 6.0 Hz, CONH<u>CH$_2$</u>), 3.830 (s, 3H), 3.574-3.511 (m, 2H), 2.818 (d, 6H, = 4.4 Hz, NH^{+}<u>CH$_3$</u>)$_2$.^{13}C NMR (101 MHz, DMSO-d$_6$) δ 163.17, 153.38, 137.69, 133.63, 133.29, 128.95, 128.84, 127.50, 126.49, 126.25, 120.67, 120.15, 112.66, 112.22, 111.28, 103.47, 56.05, 54.69, 42.47, 42.39, 41.21, 41.06. HRMS (ESI), m/z [M+H]$^{+}$, calcd for $C_{22}H_{23}ClN_4O_2$: 411.1588, found 411.1592.

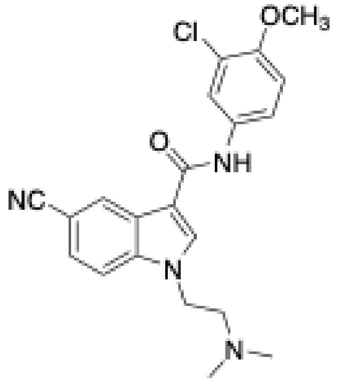

***N*-(3-chloro-4-methoxyphenyl)-5-cyano-1-(2-(dimethylamino)ethyl)-1*H*-indole-3-carboxamide (14b)**. 5% MeOH in DCM. Yield: 71% (white solid). ^{1}H NMR (400 MHz, DMSO-d$_6$) 9.936 (s, 1H, CONH), 8.585 (d, 1H, J = 1.2 Hz), 8.449 (s, 1H), 7.927 (d, 1H, J = 2.8 Hz), 7.843 (d, 1H, J = 8.8 Hz), 7.628 (dt, 2H, J = 2.8, 9.2 Hz), 7.161 (d, 1H, J = 9.2 Hz), 4.405 (t, 2H, J = 6.0 Hz), 3.846 (s, 3H), 2.670 (t, 2H, J = 6.4 Hz), 2.195 (s, 6H). HRMS (ESI), m/z [M+H]$^{+}$, calcd for $C_{21}H_{21}ClN_4O_2$: 397.1431, found 397.1433.

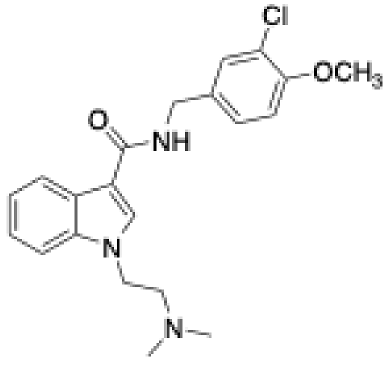

***N*-(3-chloro-4-methoxybenzyl)-1-(2-(dimethylamino)ethyl)-1*H*-indole-3-carboxamide (14c)**. 5% MeOH in DCM. Yield: 37% (white solid). ^{1}H NMR (400 MHz, DMSO-d_6) δ 8.398 (t, 1H, J = 5.6 Hz), 8.160 (d, 1H, J =8.0 Hz), 8.07 (s, 1H), 7.536 (d, 1H, J = 8.4 Hz), 7.387 (d, 1H, J = 2.0 Hz), 7.282 (dd, 1H, J = 2.0, 8.0 Hz), 7.197 (dt, 1H, J = 1.2, 7.2 Hz), 7.150-7.090 (m (dt and dd overlapping), 2H), 4.398 (d, 2H, J = 6.0 Hz), 4.279 (t, 2H, 6.4 Hz), 3.824 (s, 3H), 2.626 (t, 2H, J = 6.4 Hz), 2.182 (s, 6H). HRMS (ESI), m/z $[M+H]^+$, calcd for $C_{21}H_{24}ClN_3O_2$: 386.1365, found 386.1641.

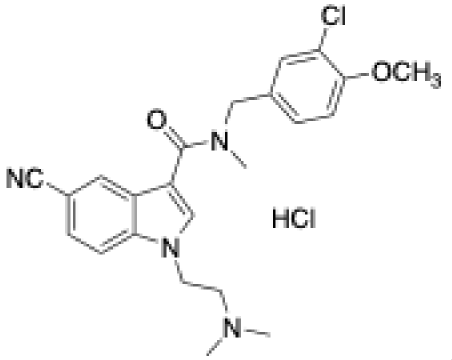

***N*-(3-chloro-4-methoxybenzyl)-5-cyano-1-(2-(dimethylamino)ethyl)-*N*-methyl-1*H*-indole-3-carboxamide hydrochloride (14d)**. 5% MeOH in DCM. Yield: 38% (colorless oil). The final product was converted into its hydrochloride form using 2.0 N HCl in diethyl ether. (400 MHz, DMSO-d_6) δ 10.896 (s, 1H, $\underline{NH}^+(CH_3)_2$), 8.333 (s, 1H), 8.166 (s, 1H), 7.900 (d, 1H, J = 8.8 Hz), 7.672 (d, 1H, J = 8.8 Hz), 7.369 (s, 1H), 7.272 (d, 1H, J = 7.6 Hz), 7.140 (d, 1H, J = 8.0 Hz), 4.740 (t, 2H, J = 6.4 Hz), 4.676 (s, 2H, $CONCH_3\underline{CH_2}Ph$), 3.842 (s, 3H), 3.505 (s, 2H), 3.325 (s, 6H), 2.774 (s, 3H, $CON\underline{CH_3}CH_2Ph$). HRMS (ESI), m/z $[M+H]^+$, calcd for $C_{23}H_{25}ClN_4O_2$: 425.1744, found 425.1740.

3. LC/MS analysis of compounds 5a-k, 10, and 14a-d.

LC/MS analysis of all final compounds was done on a Nexera-*i* LC-2040C Plus liquid chromatography system coupled to an LCMS-2020 single quadruple mass spectrophotometer (Shimadzu). The column used was 100x2.1mm C18 (stainless steel, 3 µm silica 110Å pore, ACE Equivalence) and the mobile phases were solvent A (water, 0.1% formic acid) and solvent B (acetonitrile). Unless otherwise specified, the chromatographic gradient was run at a flow rate of 0.2 mL/min as follows: 0–1 minute 10% B, 1–15 minutes 10–100% B, 15–17 minutes 100% B, 17–20 minutes 10% B.

Table S2. Mass-to-charge (m/z) values of compounds **5a-k**, **10**, and **14a-d**.

**Compound**	**m/z**
5a	169.10 [indole fragment]<br>228.05 [indole fragment+CH_3CN+H_2O]$^+$
5h	169.15 [indole fragment]<br>381.05 [M+CH_3CN+H]$^+$
5i	169.15 [indole fragment]<br>361.10 [M+CH_3CN+H]$^+$
5j	299.20 [M+H]$^+$
5g	246.20 [indole fragment]<br>403.15 [M+H]$^+$
5d	211.01 [indole fragment]<br>270.05 [indole fragment+CH_3CN+H_2O]$^+$
5e	226.00 [indole fragment]<br>285.00 [indole fragment+CH_3CN+H_2O]$^+$
5f	213.10 [indole fragment]<br>272.15 [M+CH_3CN+H_2O]$^+$
5c	209.05 [indole fragment]<br>268.05 [indole fragment+CH_3CN+H_2O]$^+$
5b	197. 15 [indole fragment]<br>256.15 [indole fragment+CH_3CN+H_2O]$^+$
5k	169.25 [indole fragment]<br>419.20 [M+CH_3CN]$^+$
10	452.05 [M+H]$^+$
14a	411.20 [M+H]$^+$<br>452.25 [M+CH_3CN+H]$^+$
14b	397.15 [M+H]$^+$
14c	386.10 [M+H]$^+$
14d	425.15 [M+H]$^+$

4. Alternative docking pose of 5e.

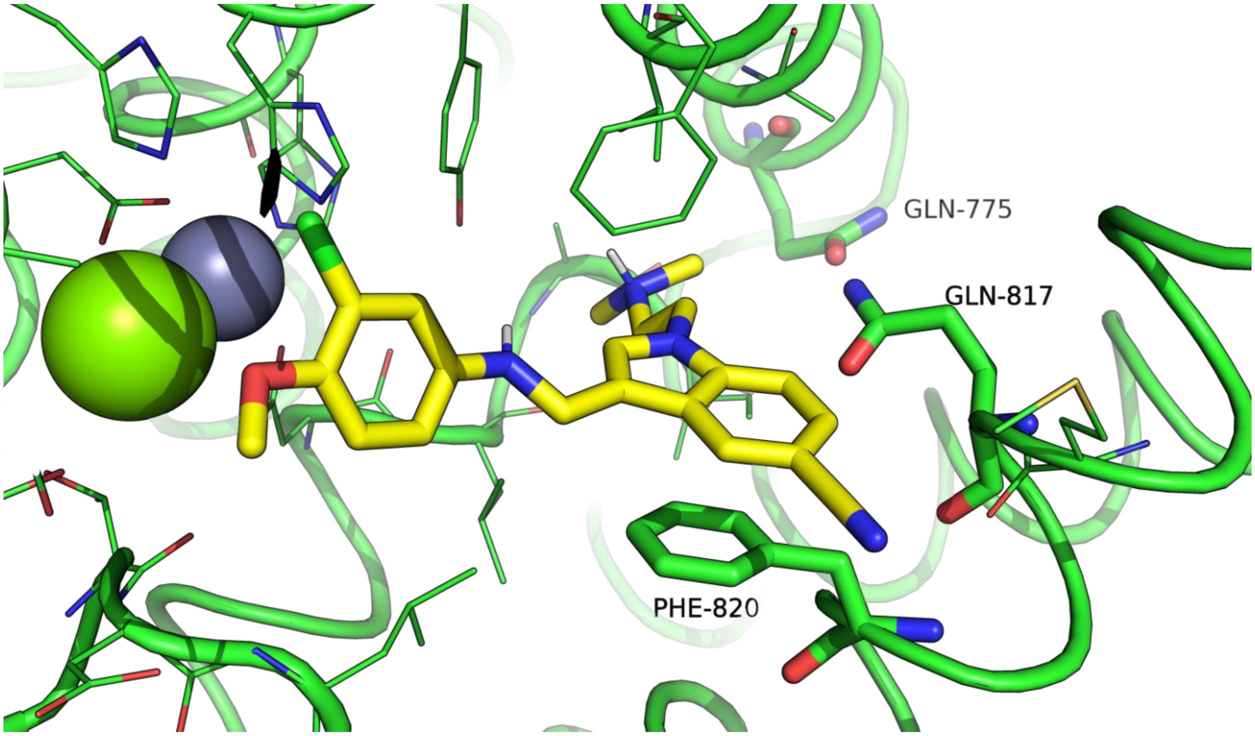

Figure S2. Docking poses of **5e** as predicted by the GOLD docking program towards the X-ray crystallography structure of PDE5 (PDB-ID: 3TGG). **5e** is shown in the conformation with the nitrile group projected towards the solvent space, and it is represented by yellow sticks. Key residues highlighted by molecular docking are shown as sticks and are labeled. Metal cations are shown as grey (Zn) and green (Mg) spheres. Residues within 5 Å from the ligands are shown as lines.

5. Molecular Dynamics Simulations of compound 14a in its neutral form.

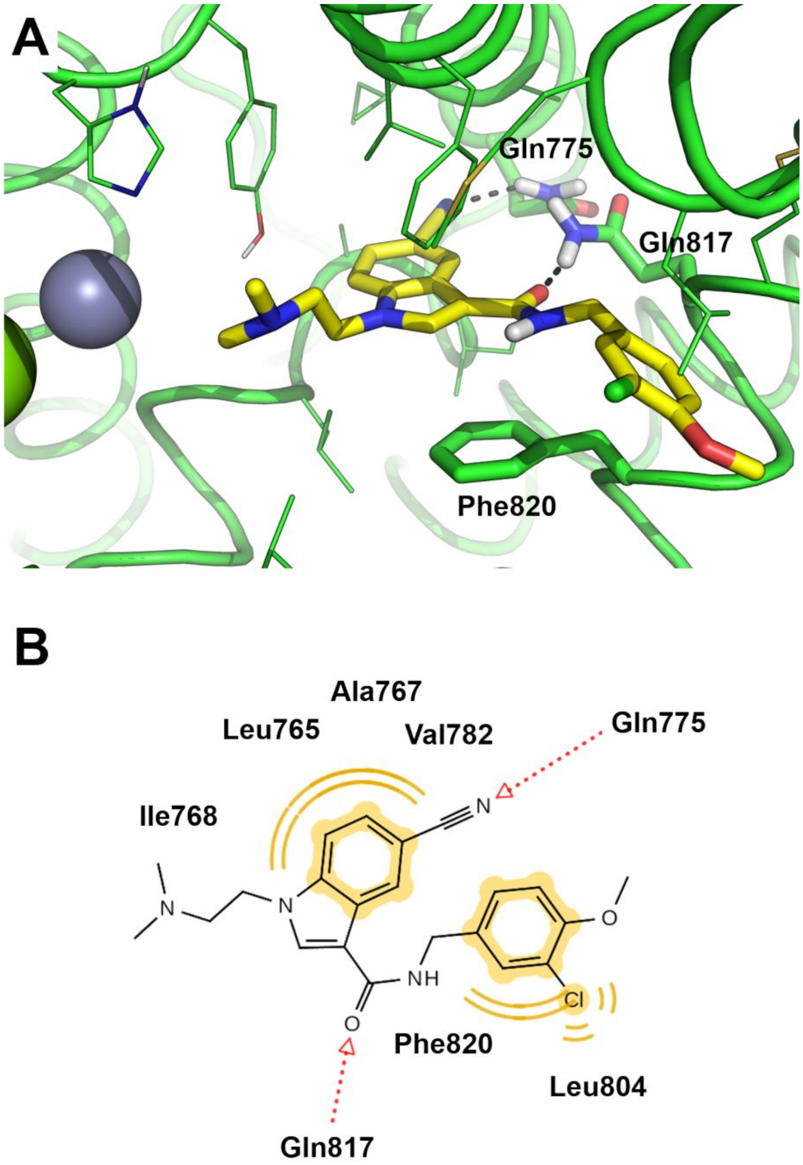

Figure S3. MD results of the interaction between the neutral form of **14a** and the PDE5 catalytic site. A) most representative frame extracted by MD trajectories describing the interaction of the neutral form of **14a** to the PDE5 site. The small molecule is shown as yellow sticks. Non-polar H atoms are omitted. Metal cations are shown as grey (Zn) and green (Mg) spheres. Residues within 5 Å from the ligand are shown as lines. Key residues for the interaction of PDE5 inhibitors studied herein are shown as sticks and are labeled. H-bond interactions are highlighted by black dashed lines. B) Two-dimensional representation of the pharmacophoric interactions of the neutral form of **14a** as depicted by the LigandScout software on the representative MD frame. Hydrophobic/aromatic interactions are highlighted by yellow curved lines. H-bonds are shown as colored dotted arrows (red = ligand is an H-bond acceptor; green = ligand is an H-bond donor); charged interactions are colored blue. To the sake of clarity, interactions with water molecules has been omitted.
