# Supplementary figures and images for: Discovery of Indole-Based PDE5 Inhibitors: Synthesis and Pharmacological Evaluation
Source: ACS Med Chem Lett. 2025 May 28;16(6):1058–65. doi: 10.1021/acsmedchemlett.5c00108 (PMC12169455; doi:10.1021/acsmedchemlett.5c00108)

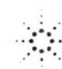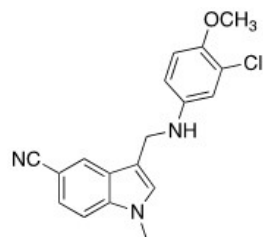

5a

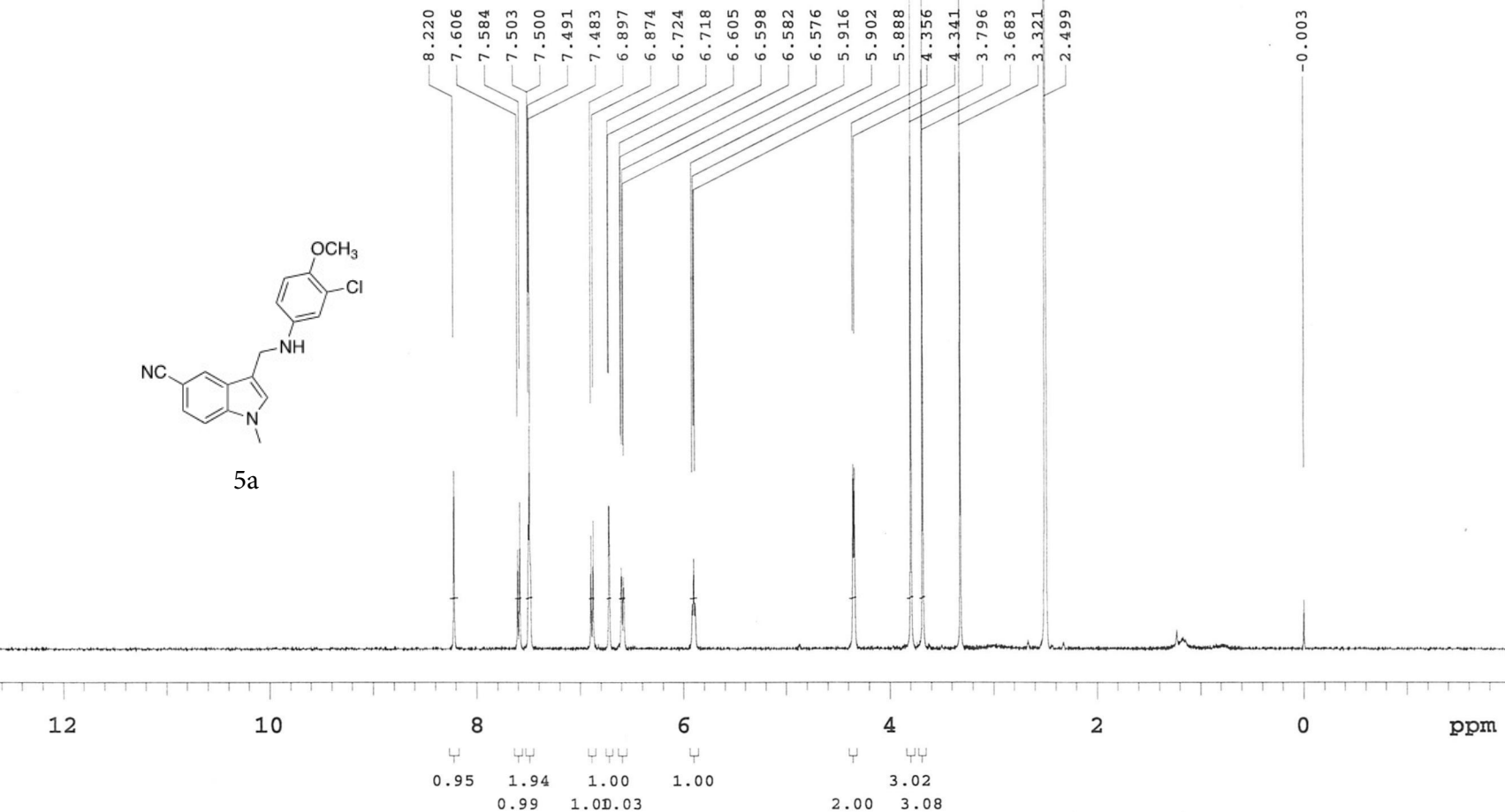

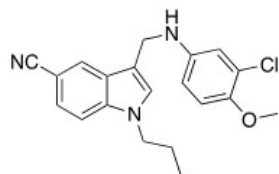

5b

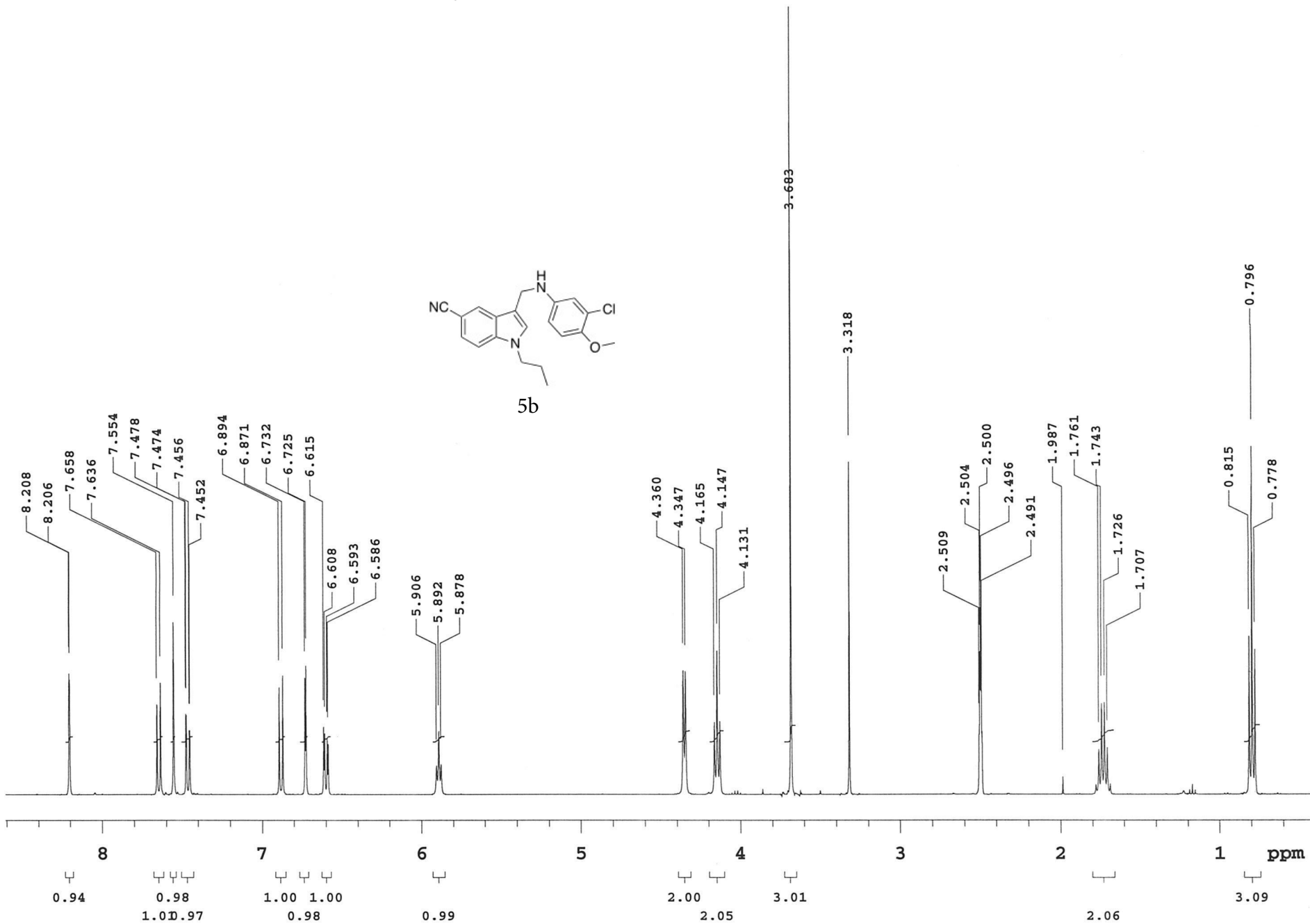

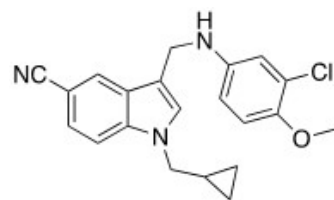

5c

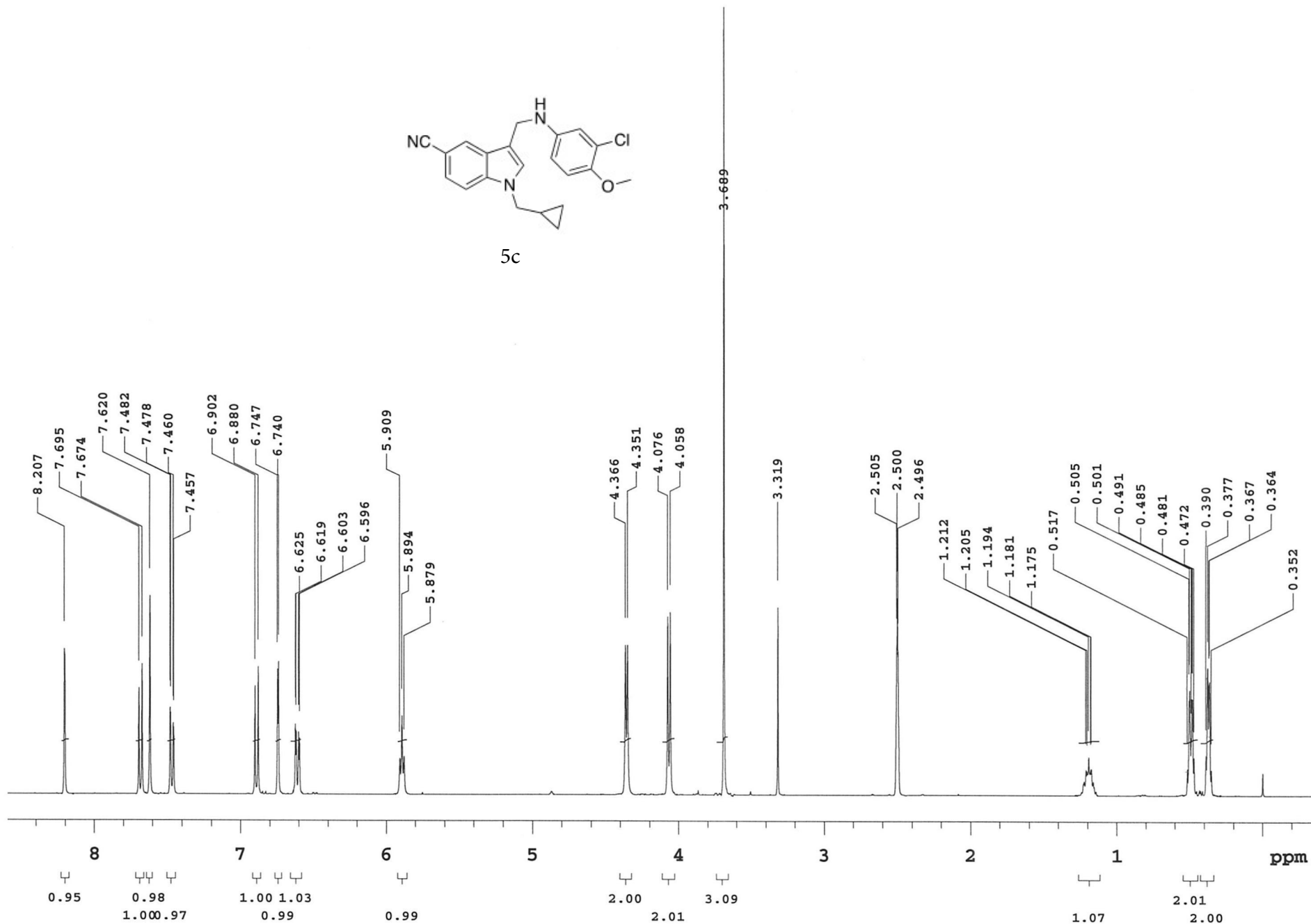

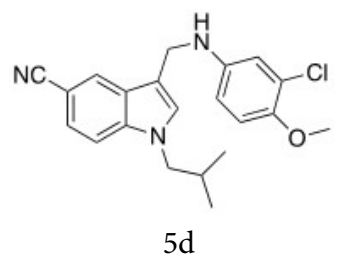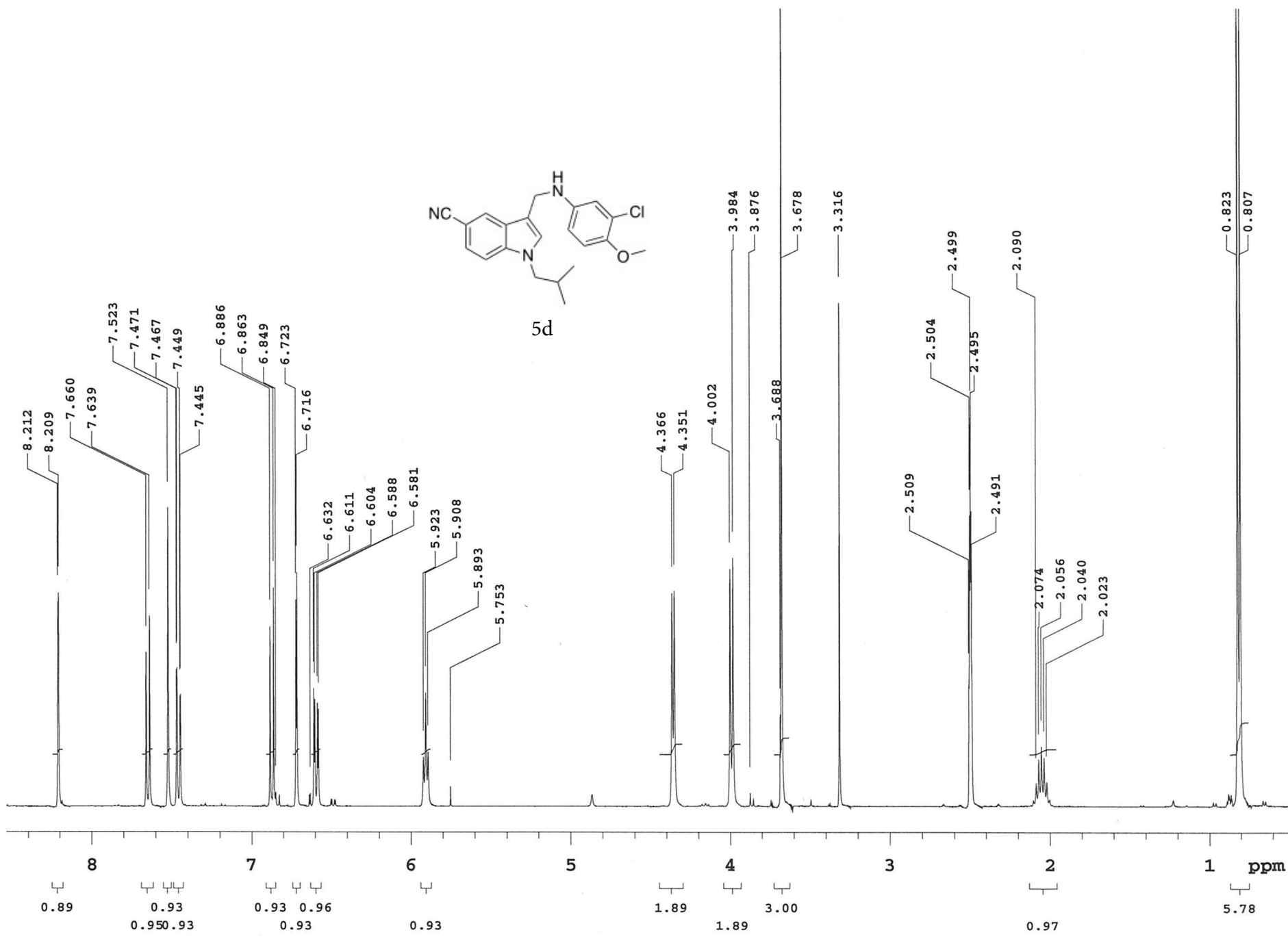

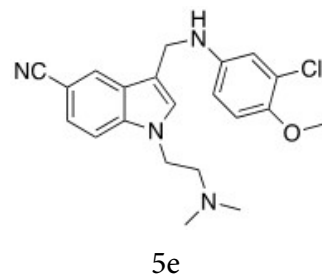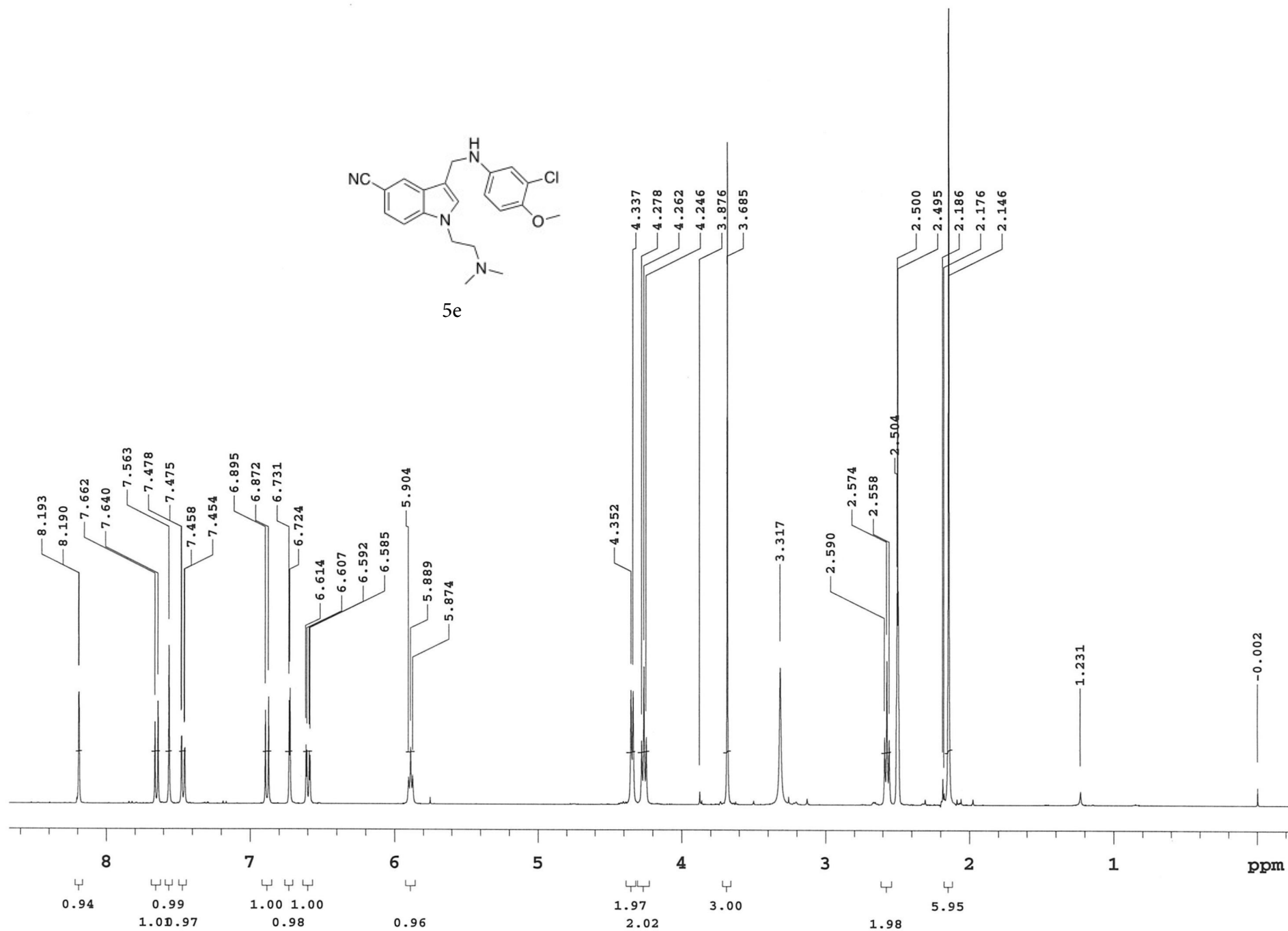

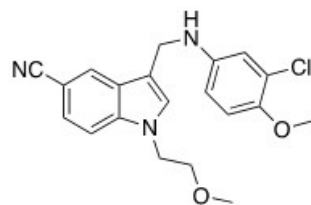

5f

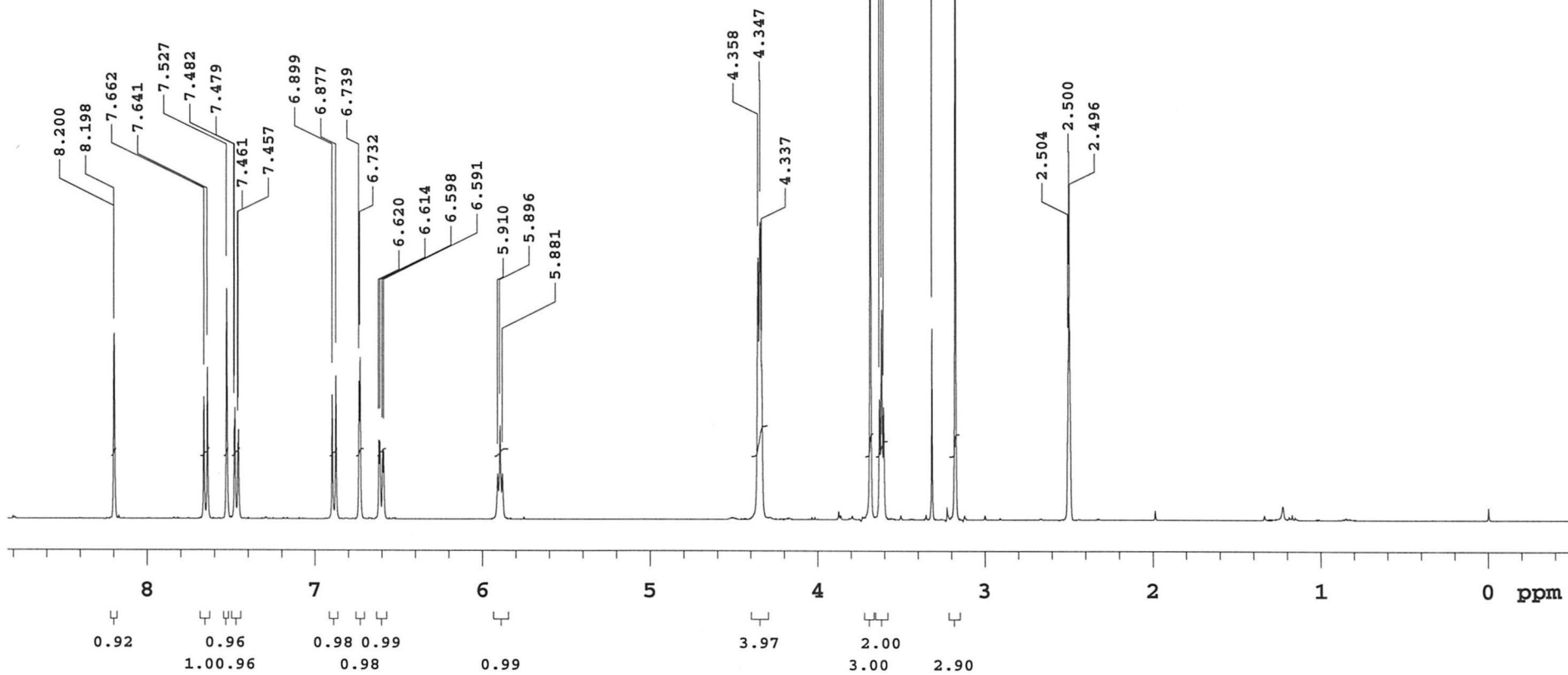

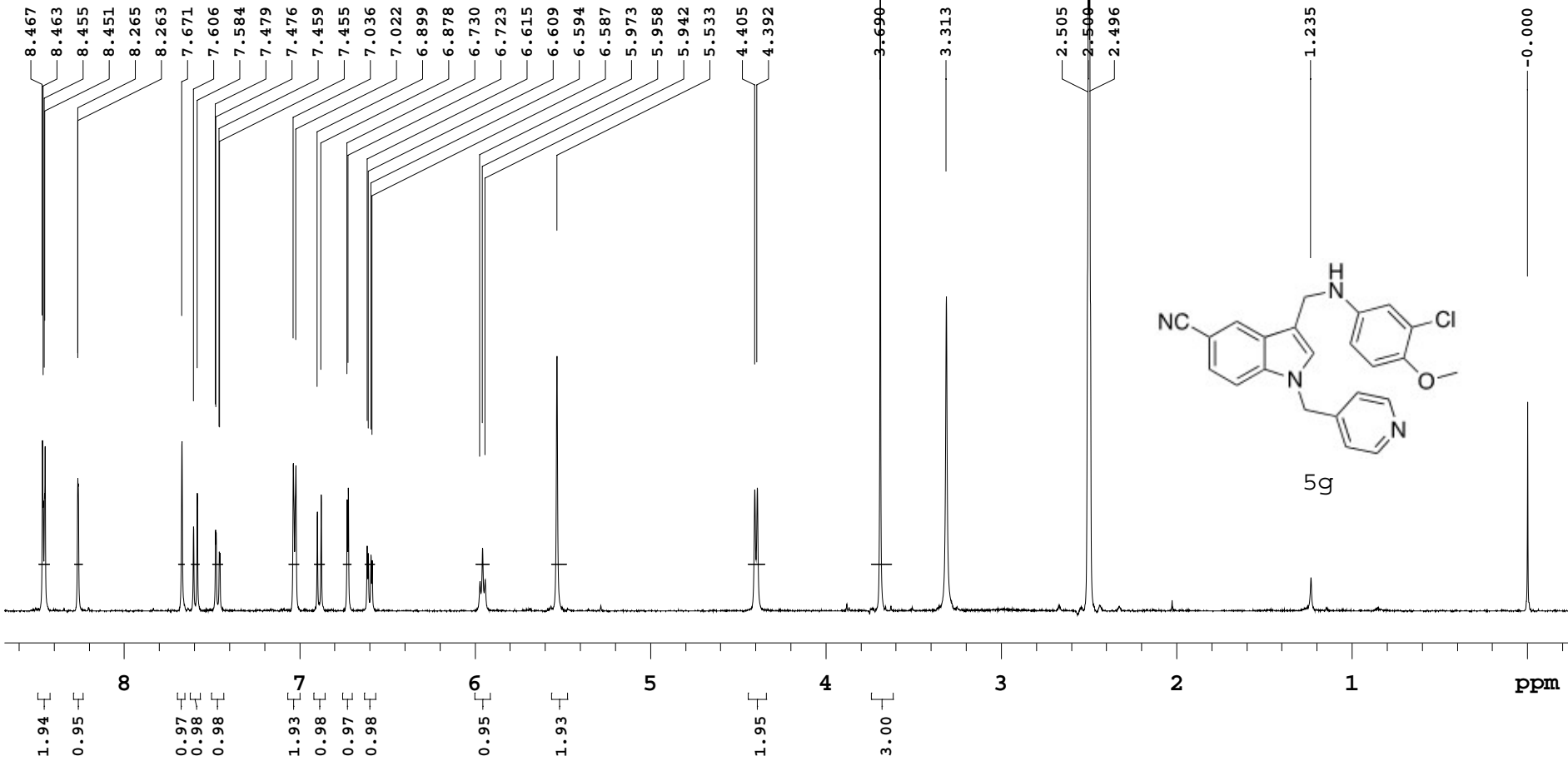

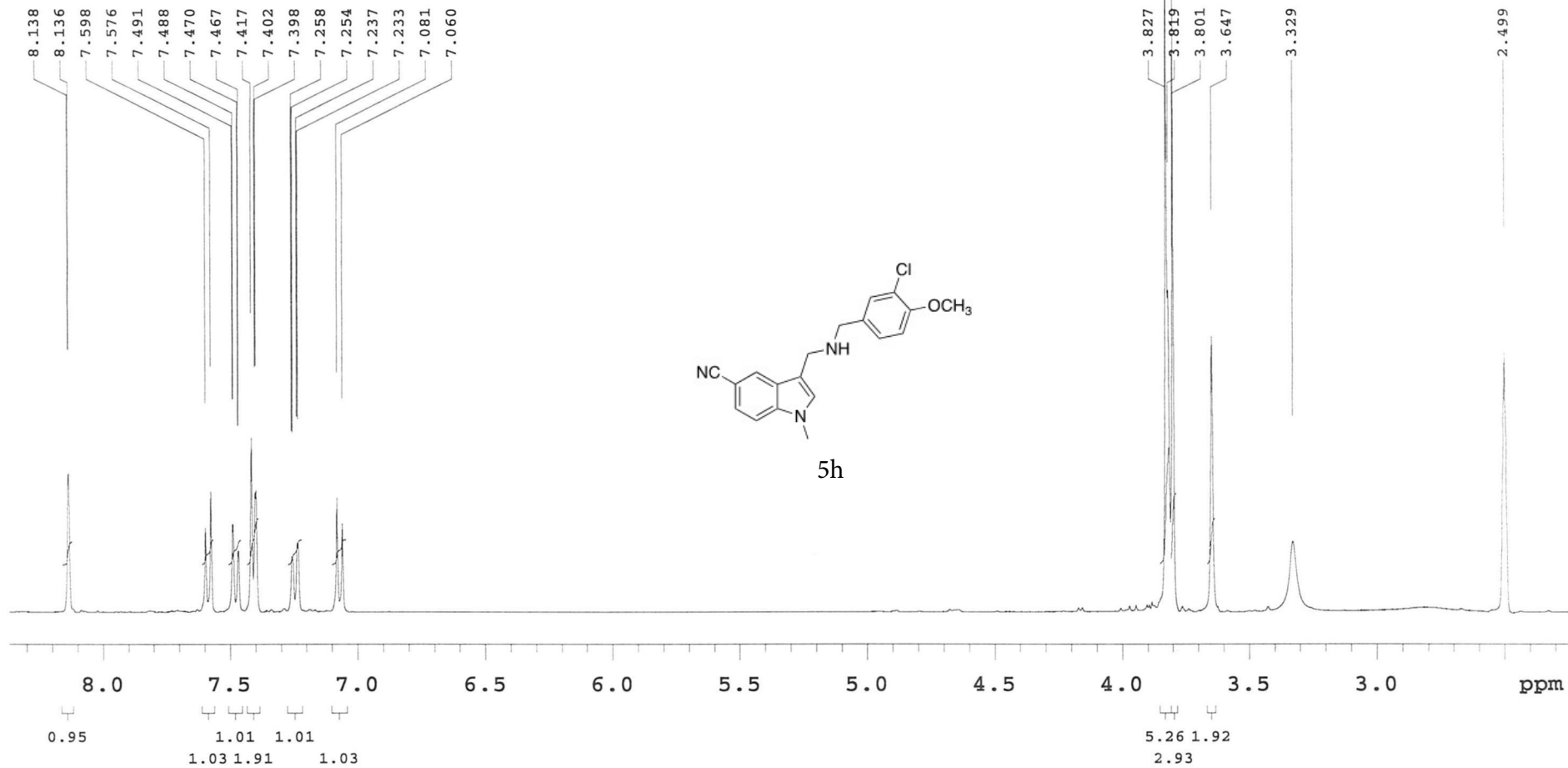

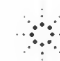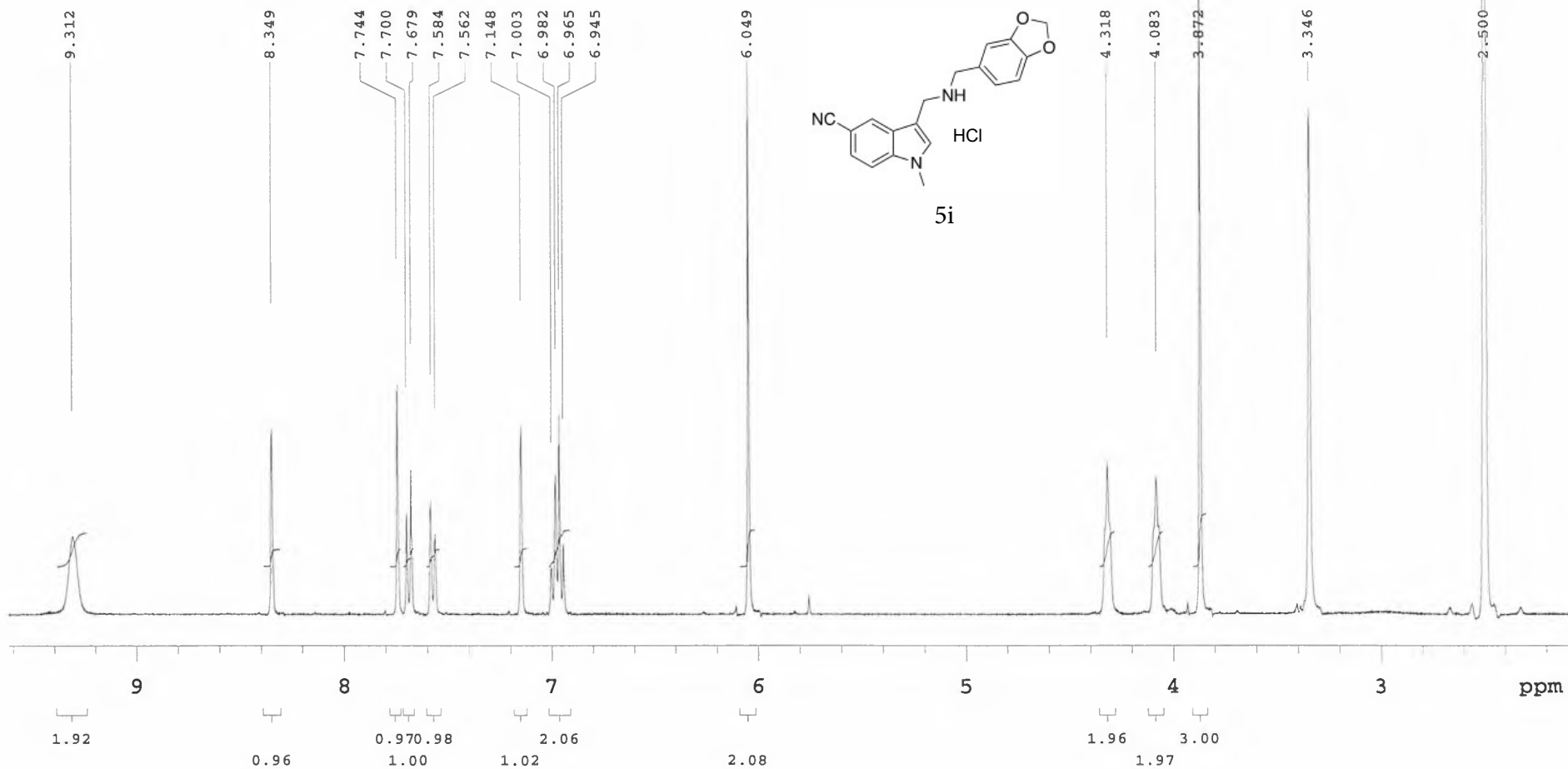

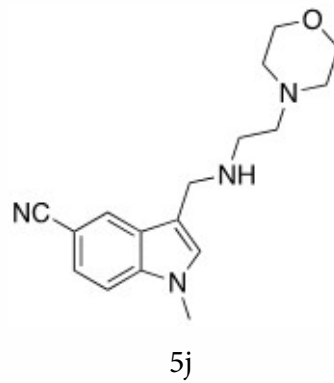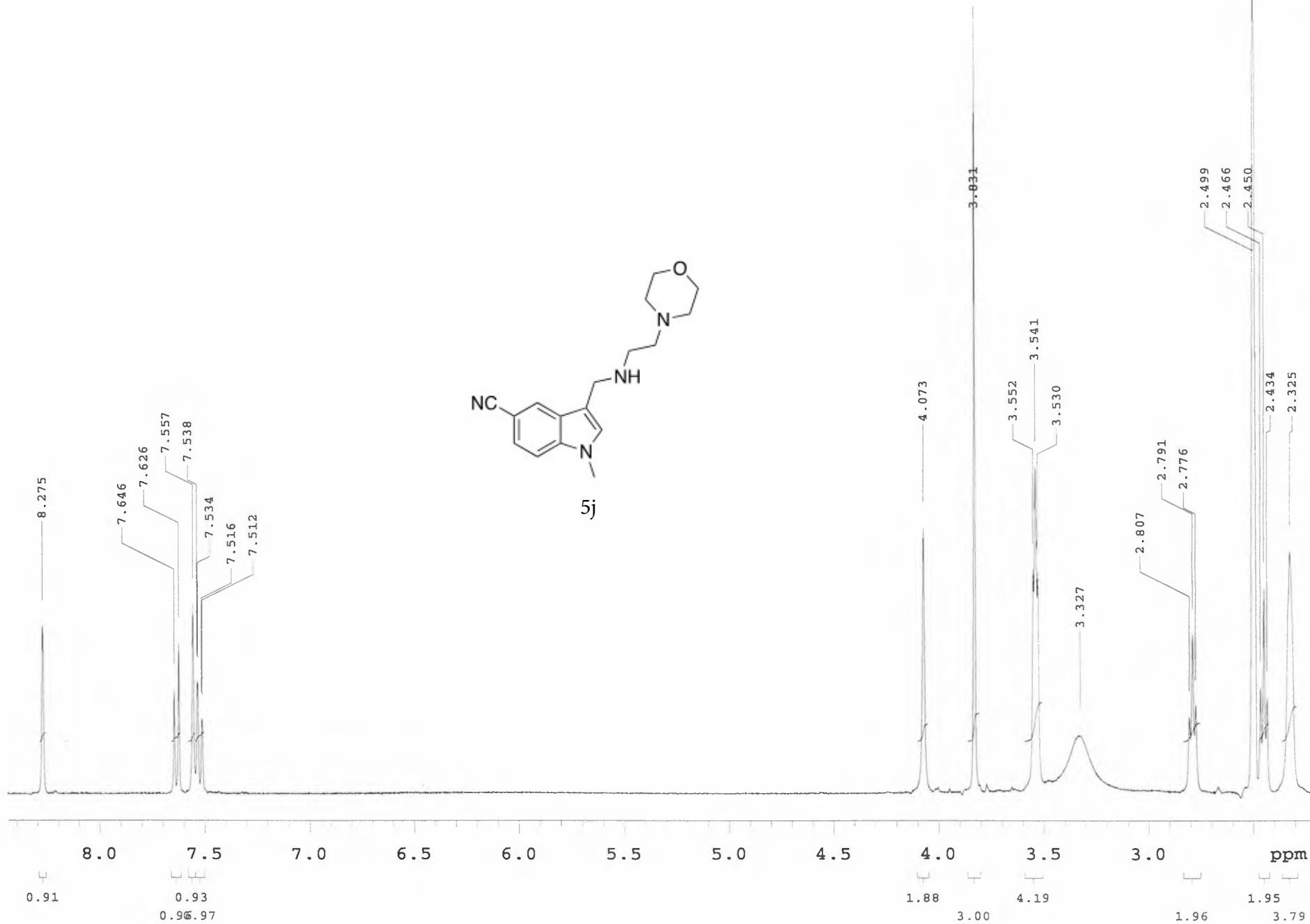

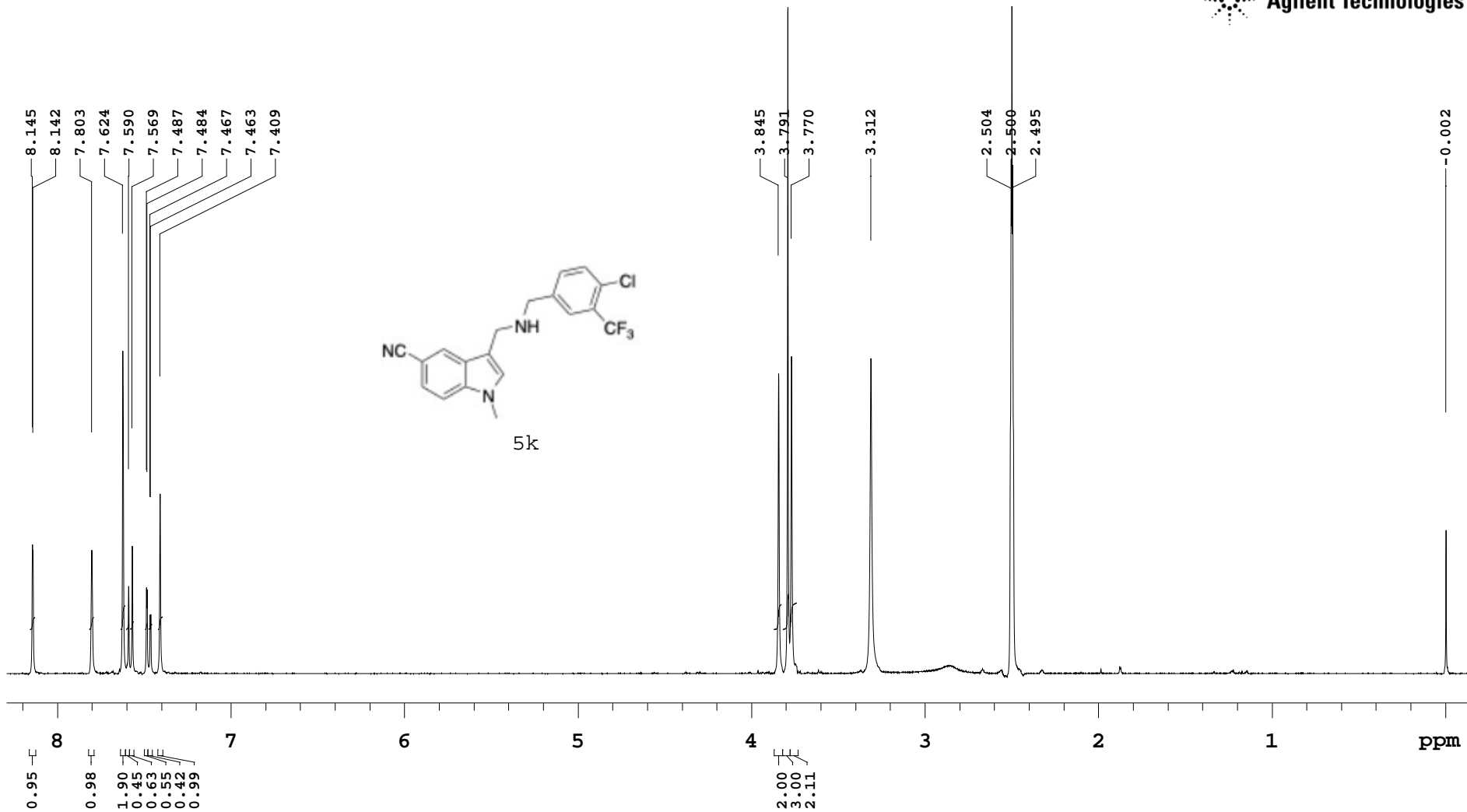

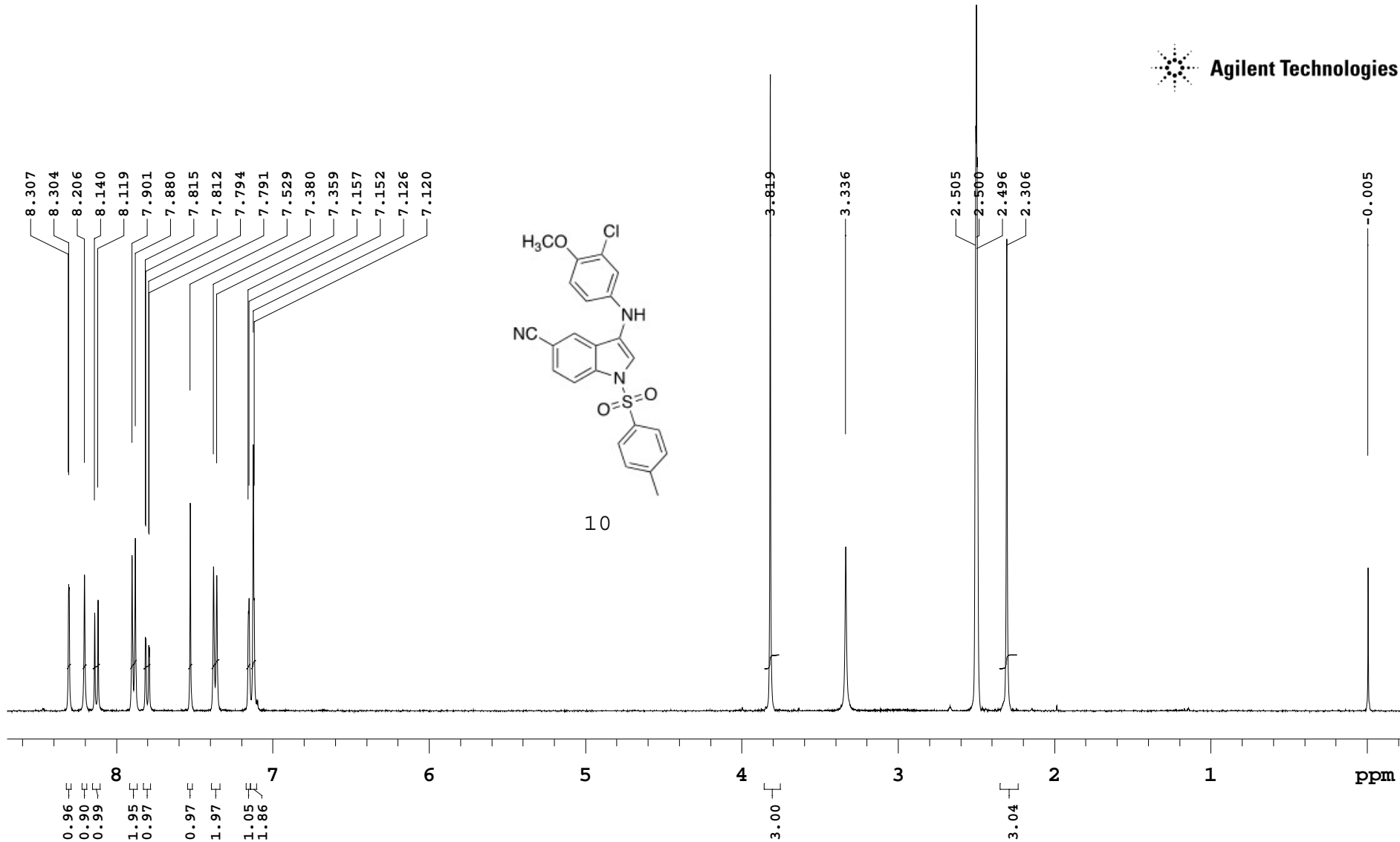

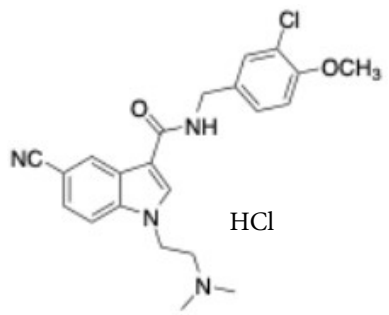

HCl

14a

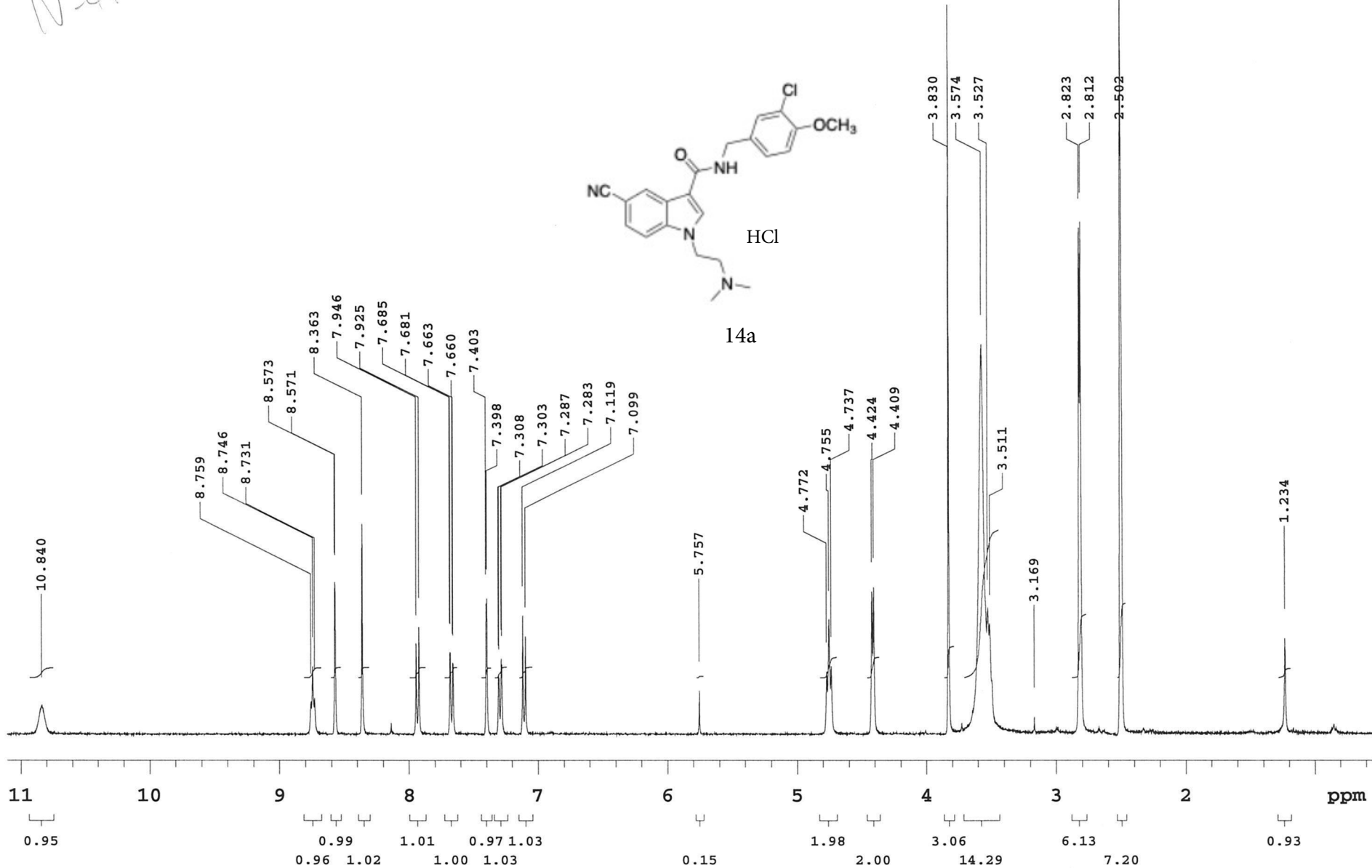

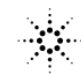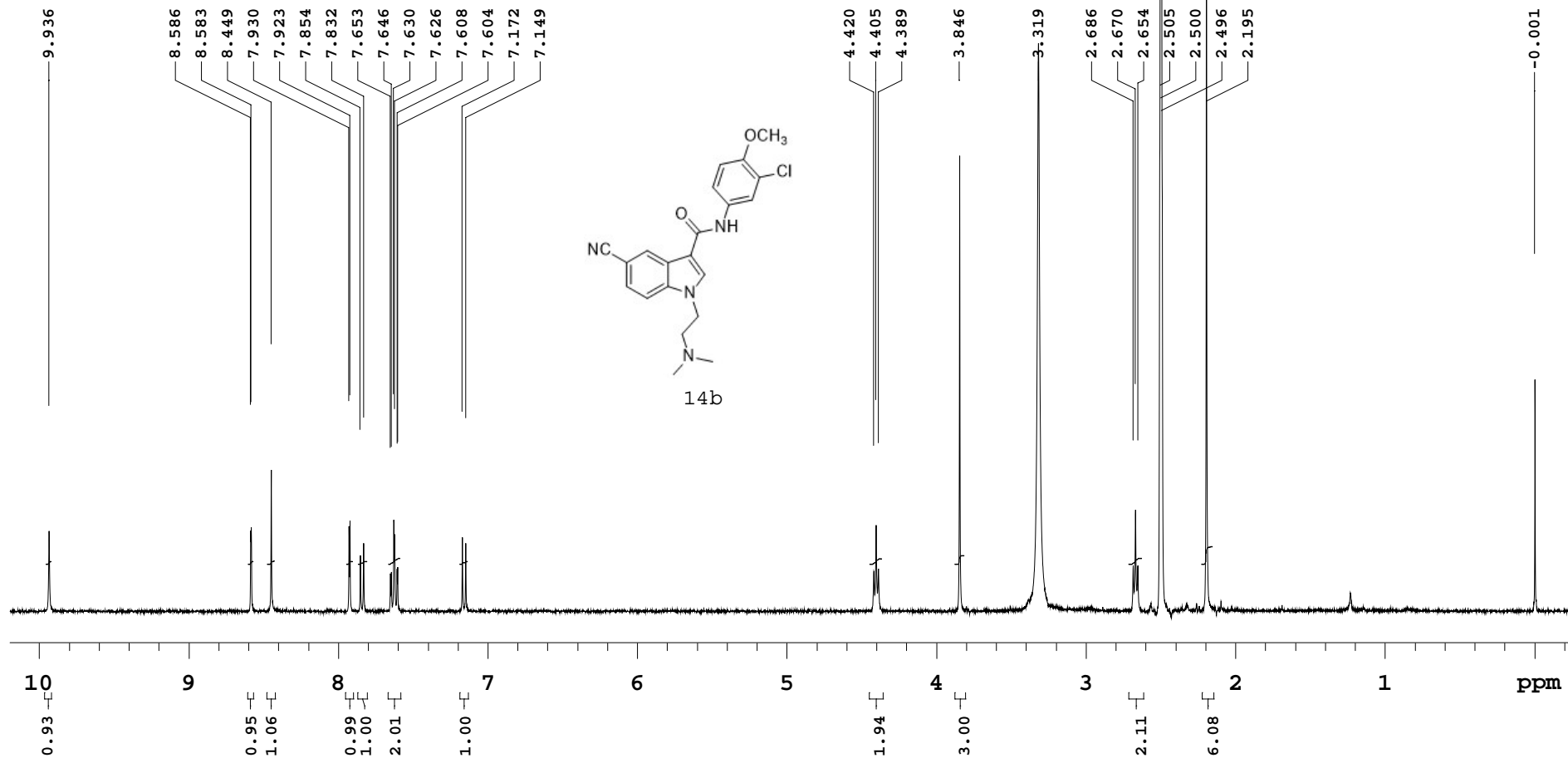

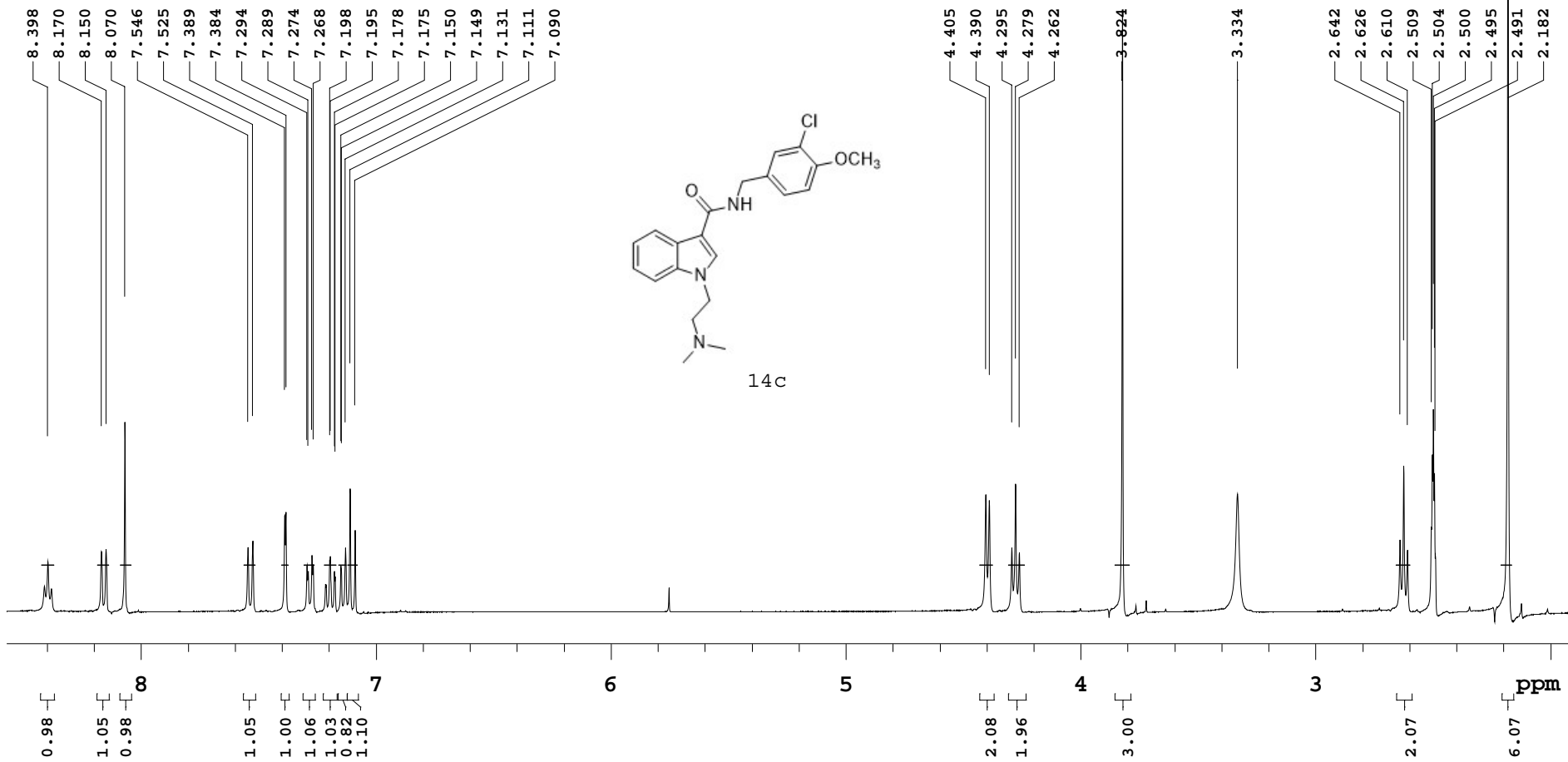

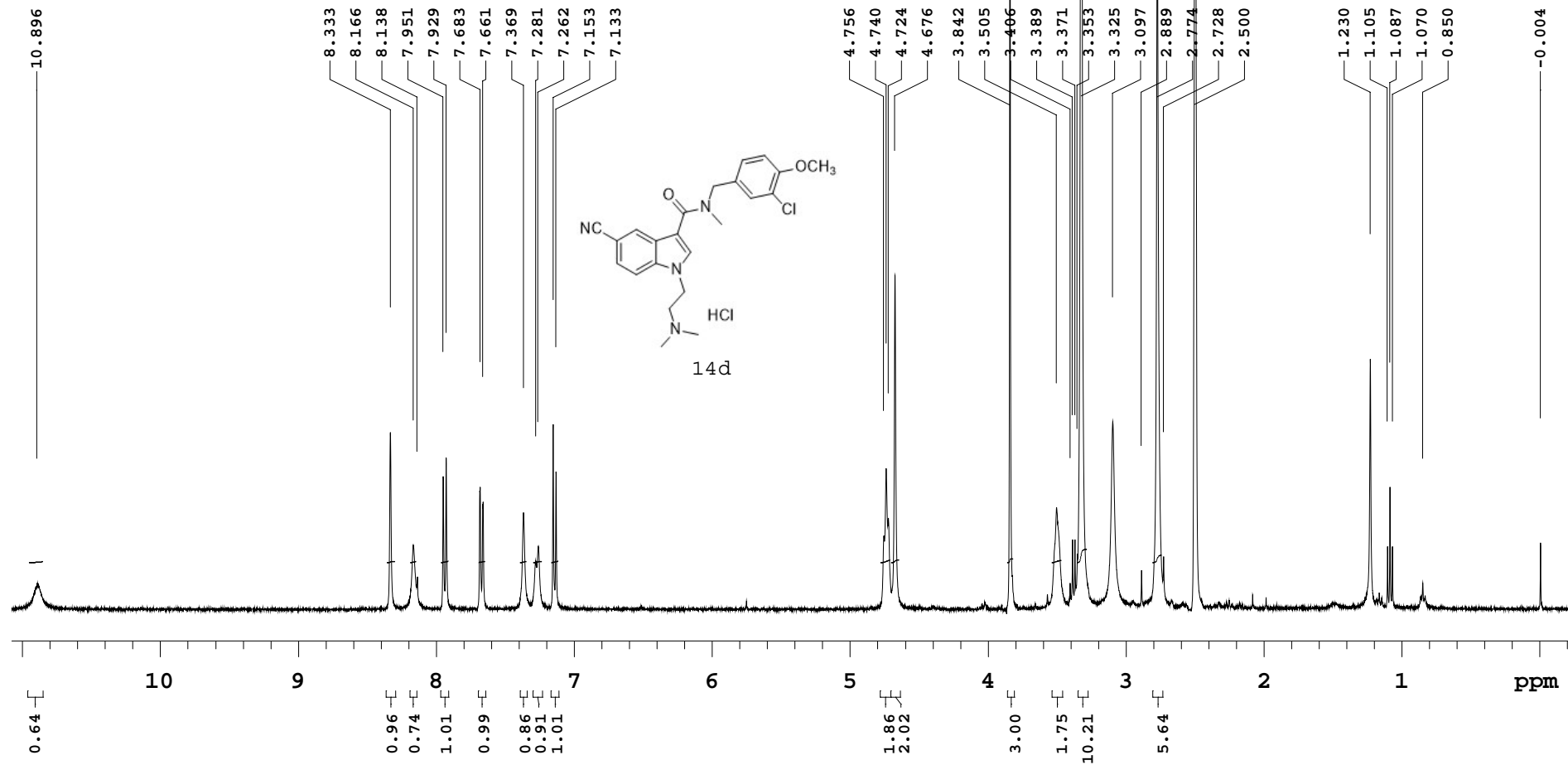

Supplement: Supplementary file 2 [file ml5c00108_si_002.pdf]

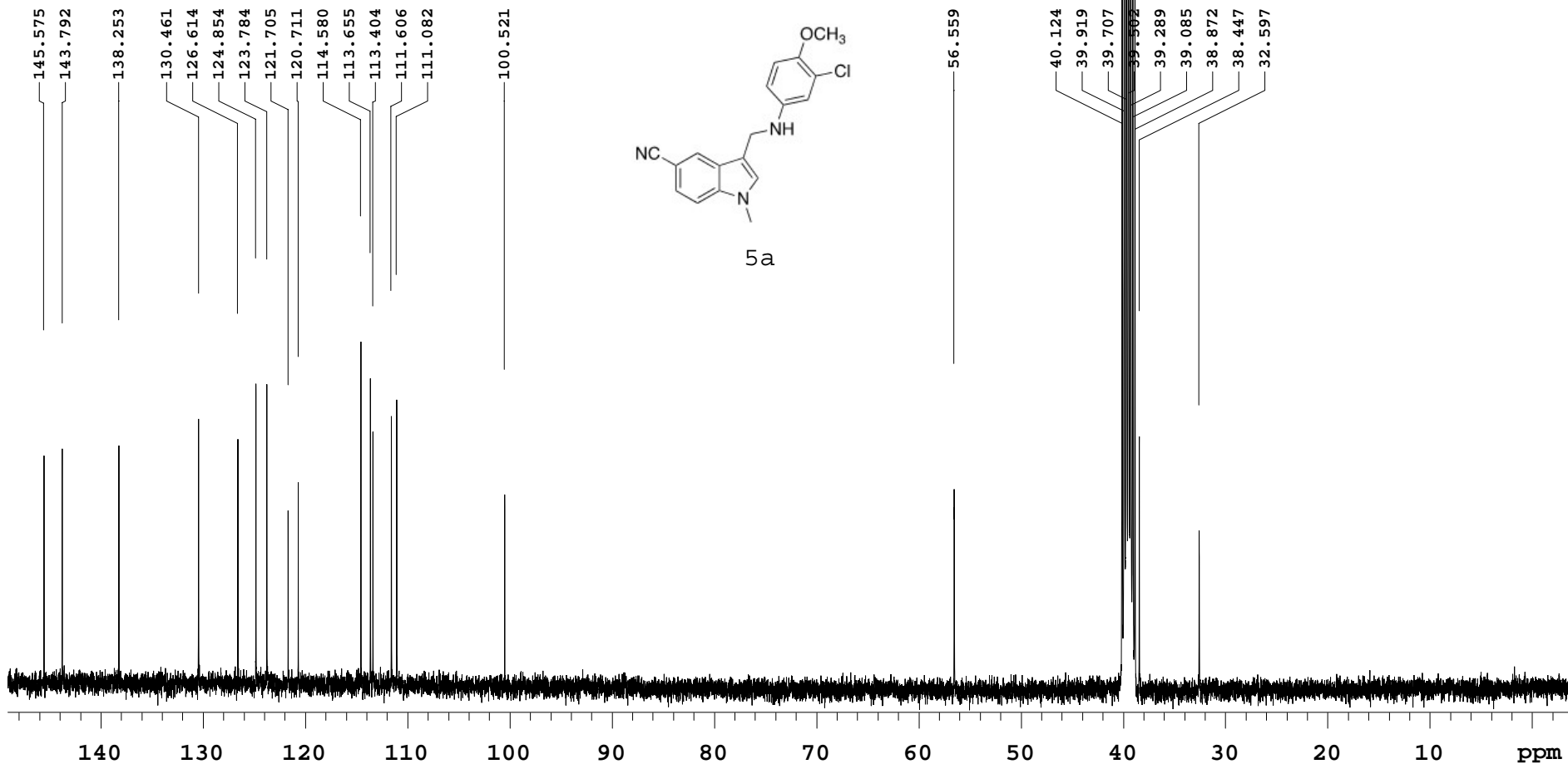

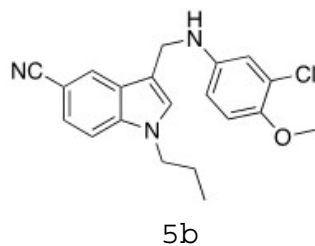

145.589  
 143.828  
 137.789  
 129.723  
 129.647  
 126.665  
 124.988  
 123.721  
 121.696  
 120.717  
 114.578  
 113.660  
 113.357  
 111.756  
 111.202  
 100.473

56.610  
 56.534  
 47.057  
 40.145  
 39.940  
 39.728  
 39.523  
 39.310  
 39.105  
 38.893  
 38.627

23.126

11.024

140 130 120 110 100 90 80 70 60 50 40 30 20 10 ppm

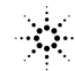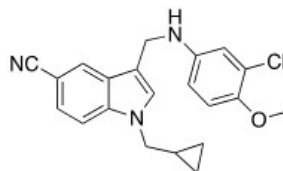

5c

145.486  
143.764

137.641

129.416  
126.616  
124.833  
123.634  
121.601  
120.615

114.476  
113.543  
113.300  
111.631  
111.176

100.394

56.462

49.588

40.035

39.830

39.618

39.413

39.201

38.996

38.783

38.540

11.392

3.547

140 130 120 110 100 90 80 70 60 50 40 30 20 10 ppm

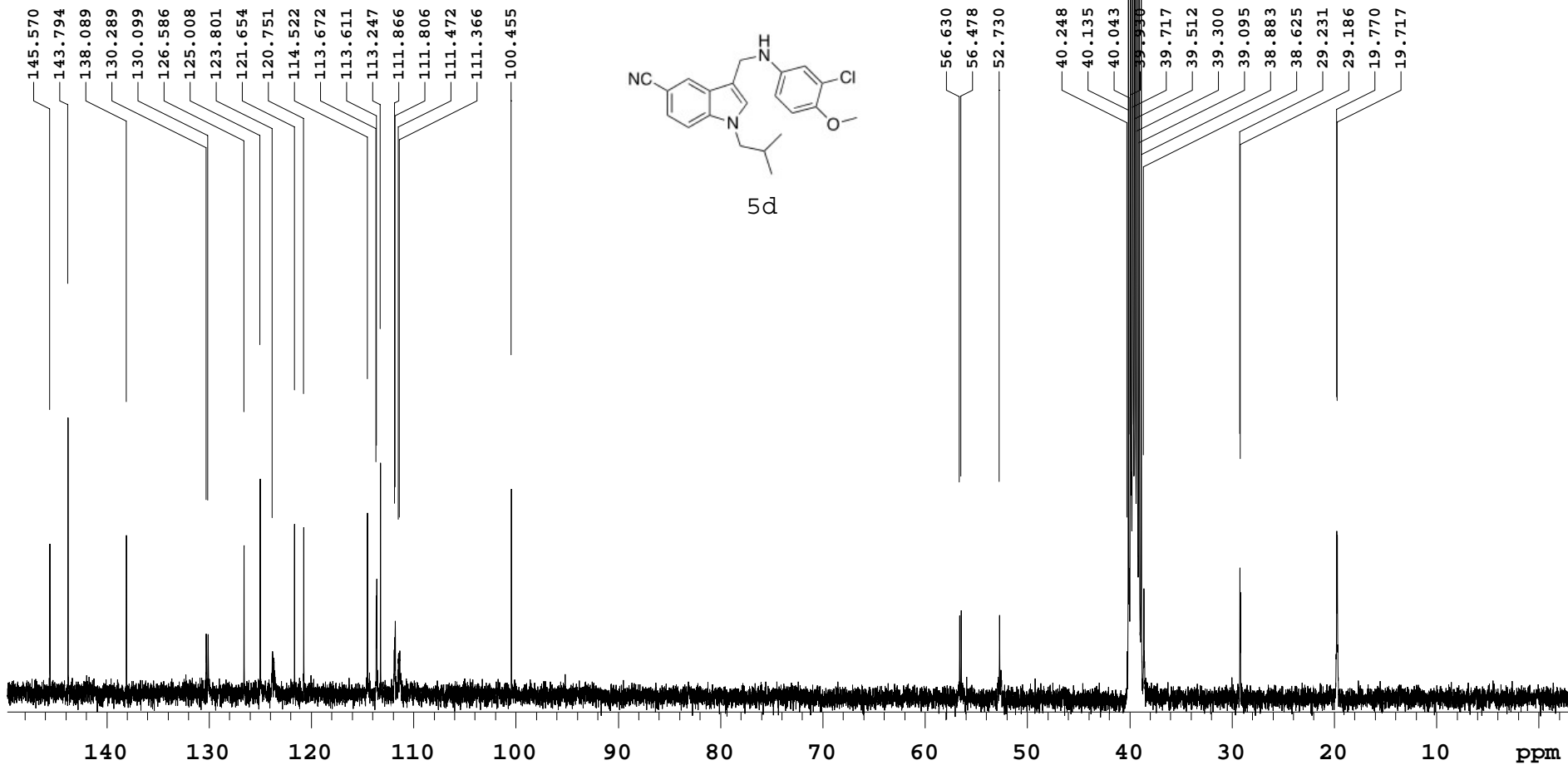

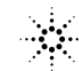

Agilent Technologies

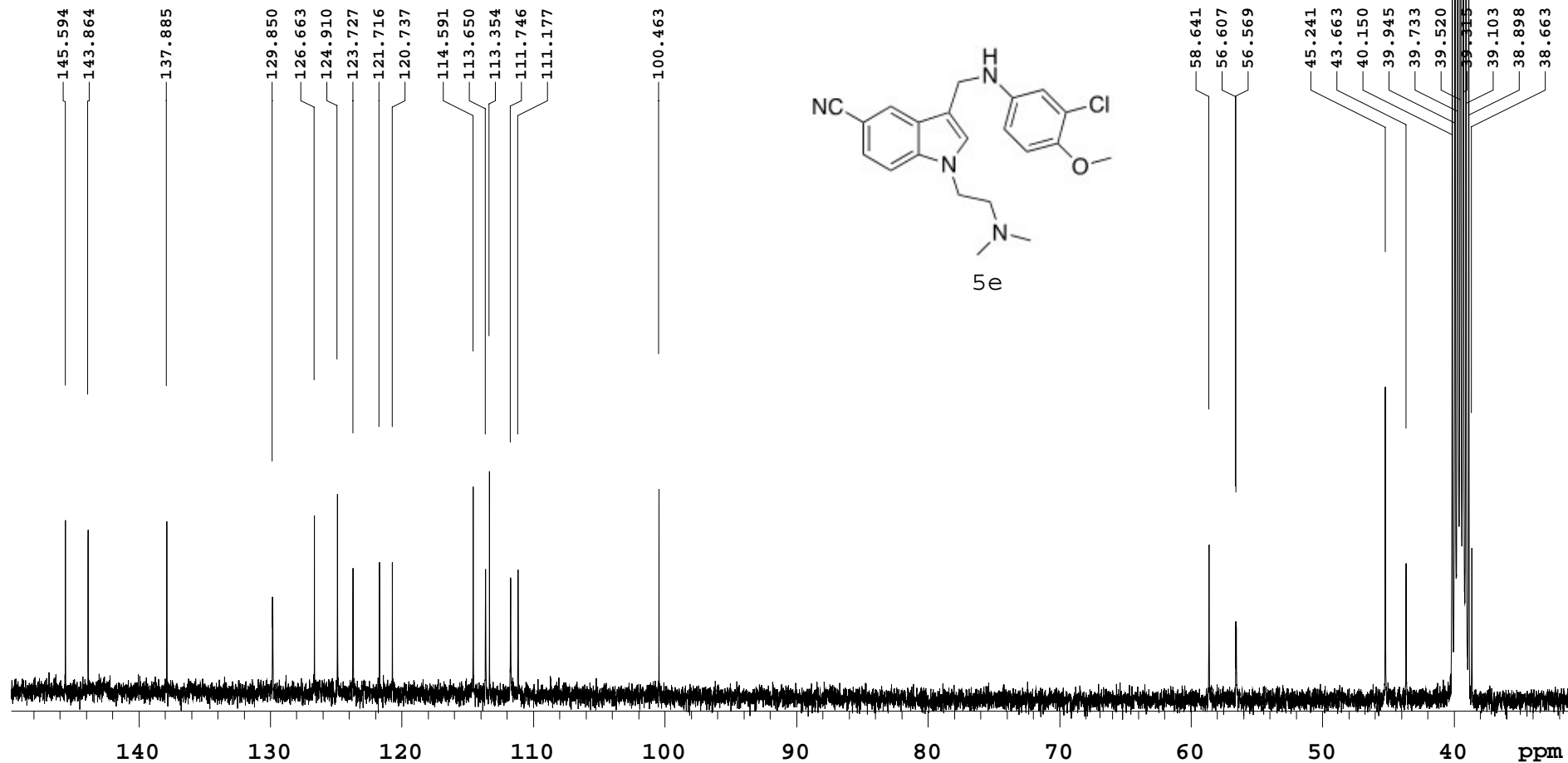

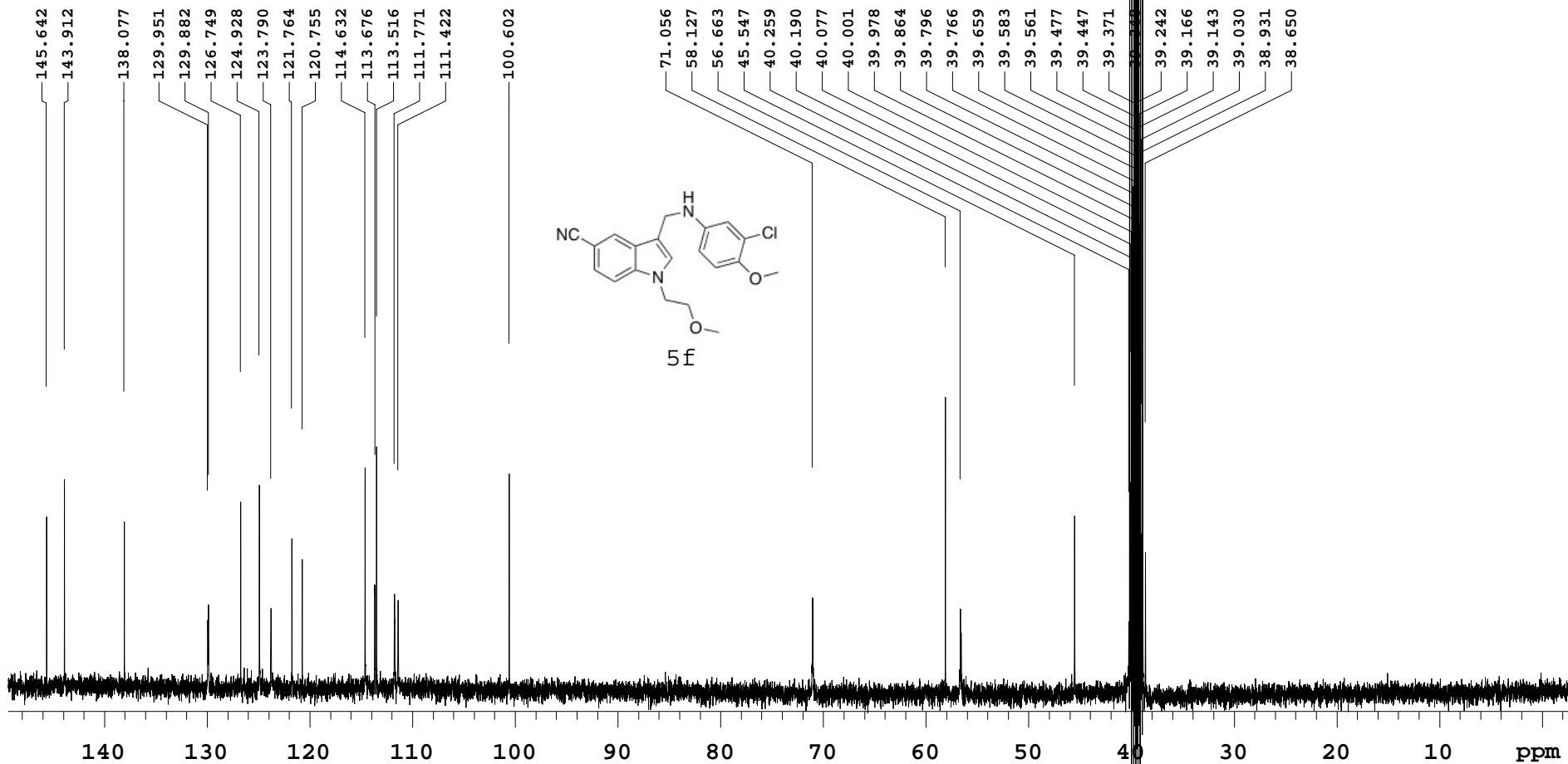

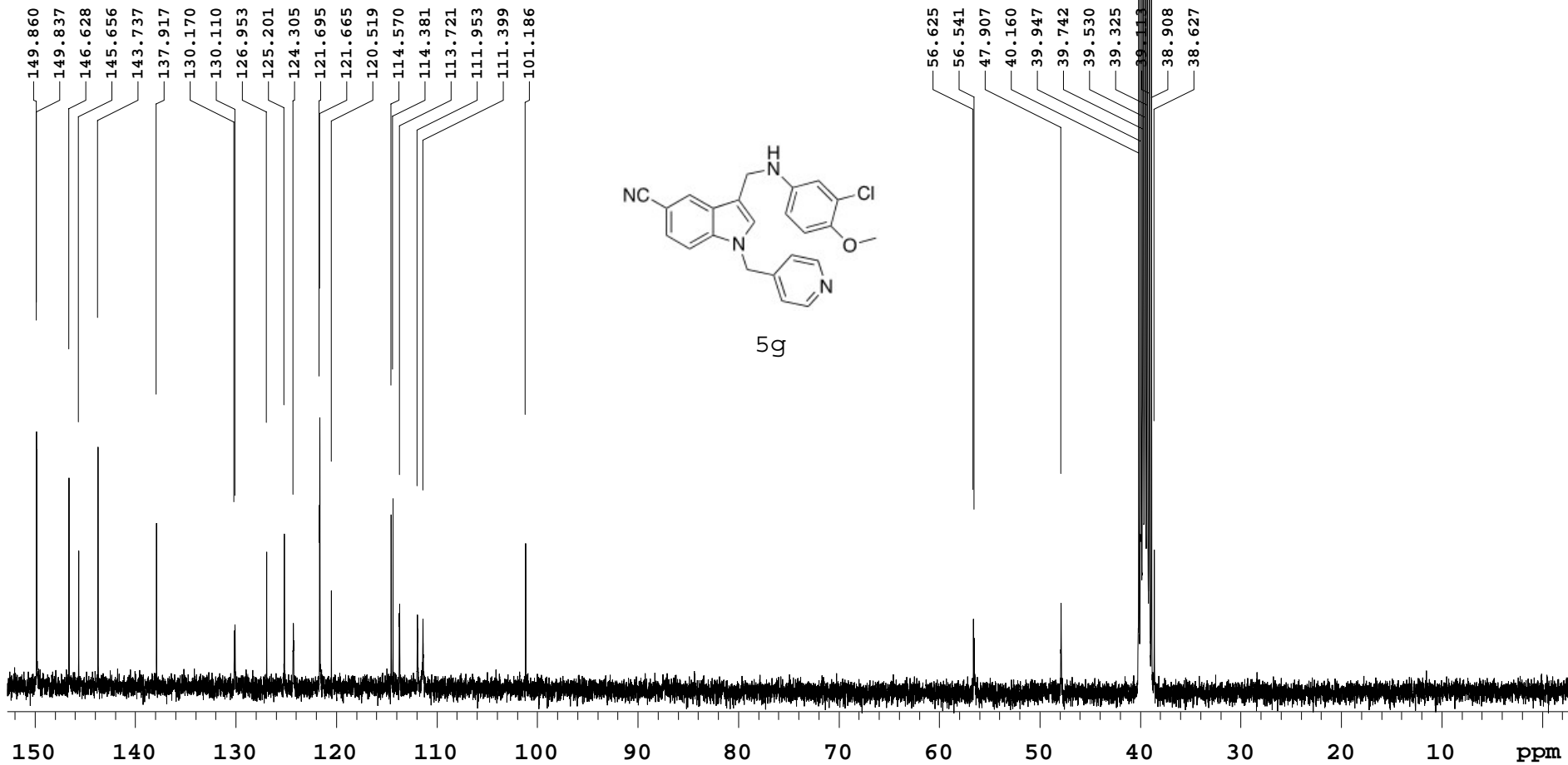

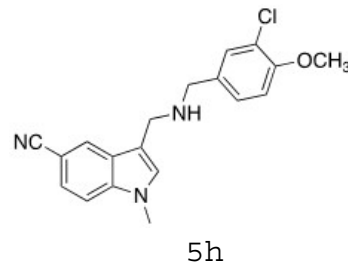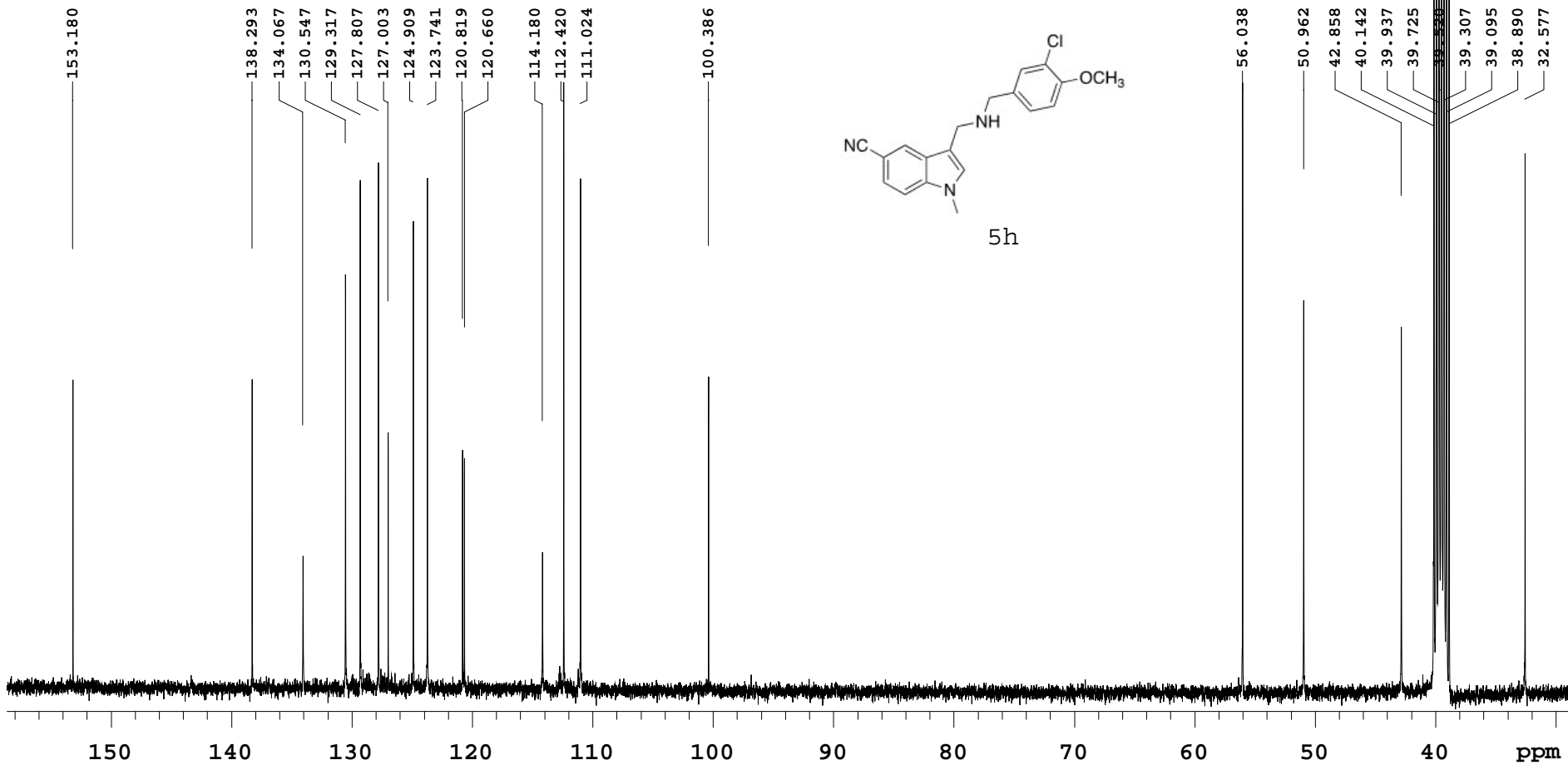

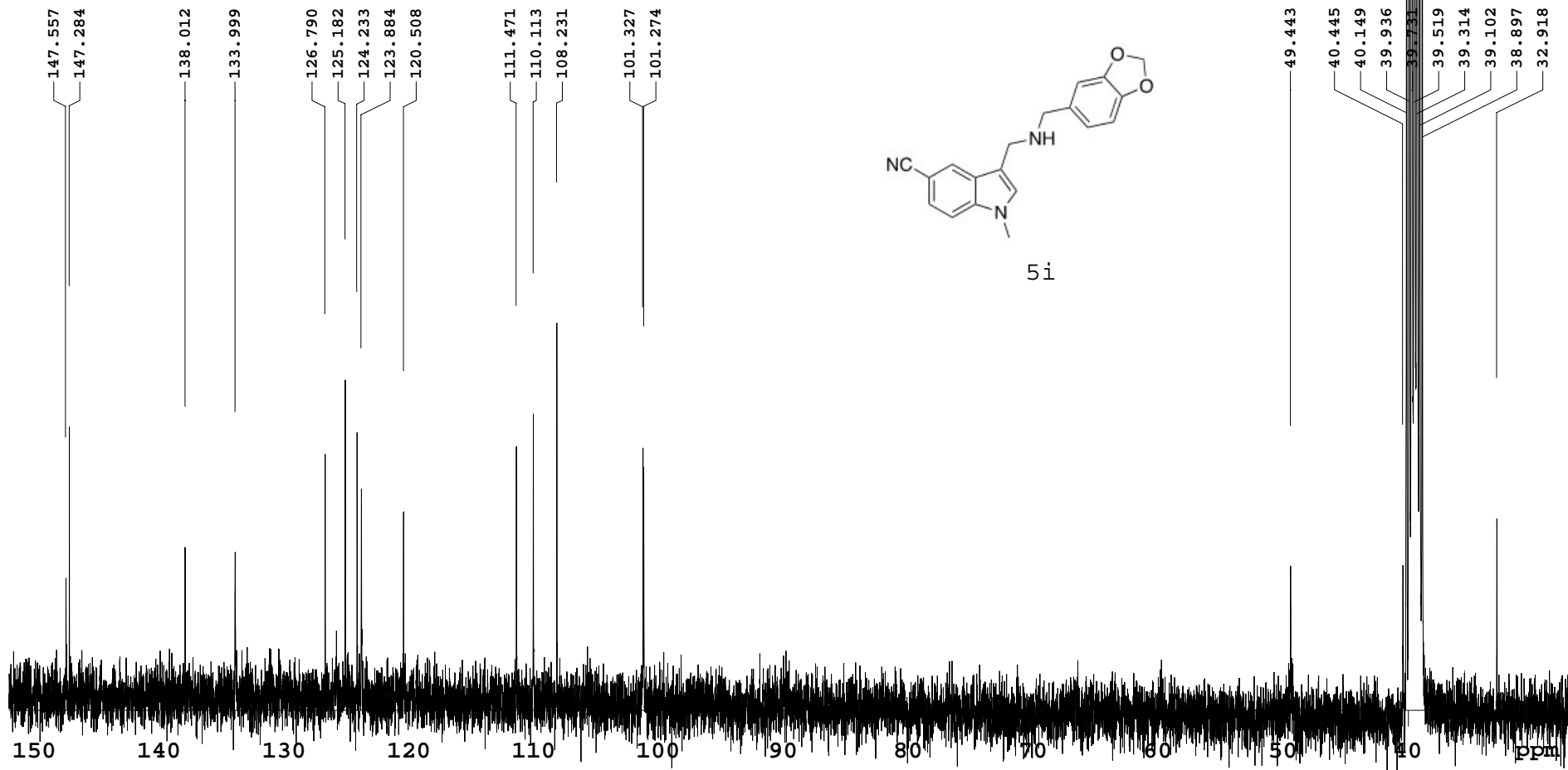

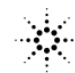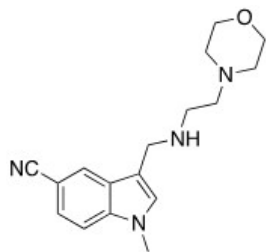

5j

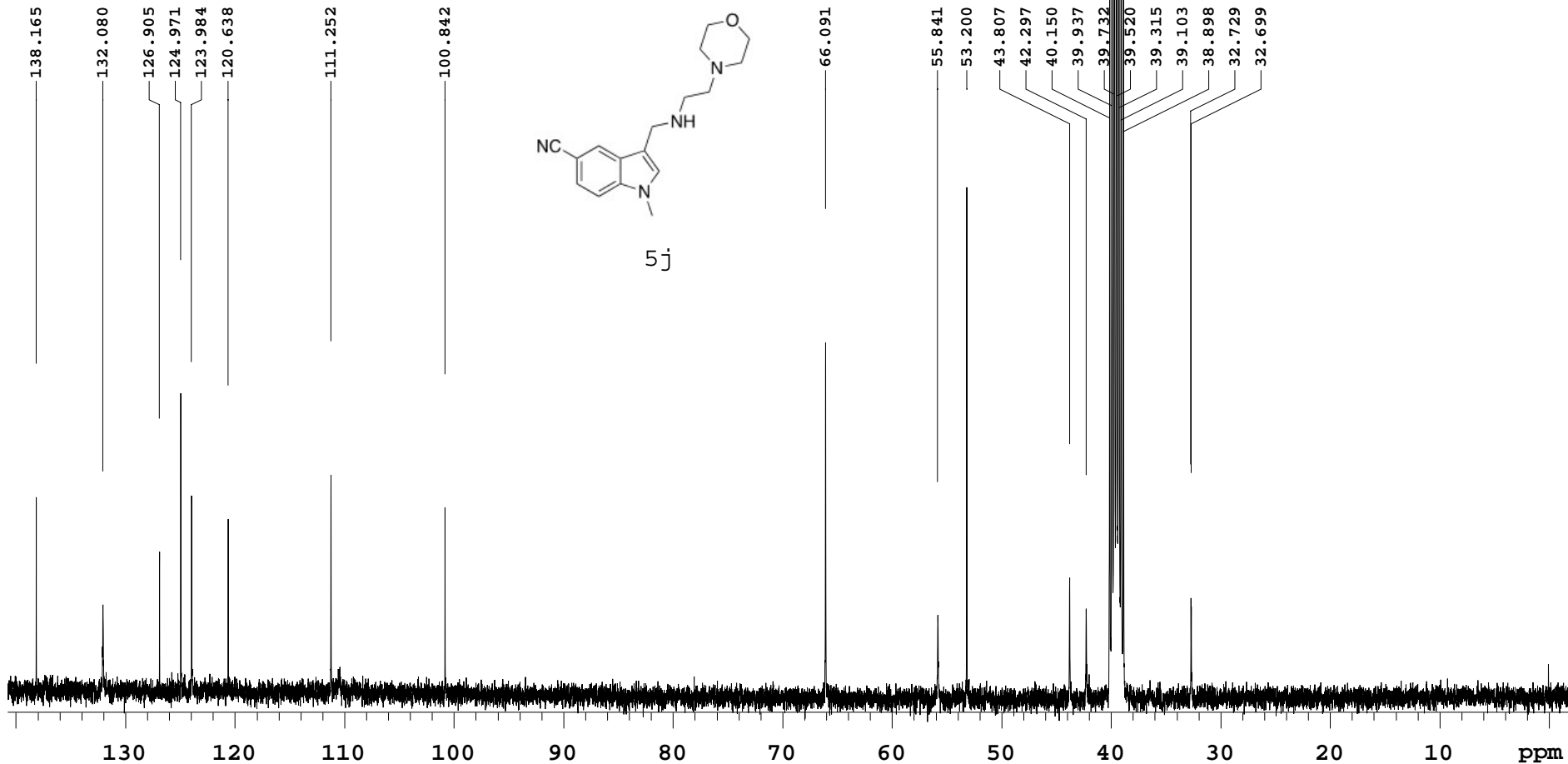

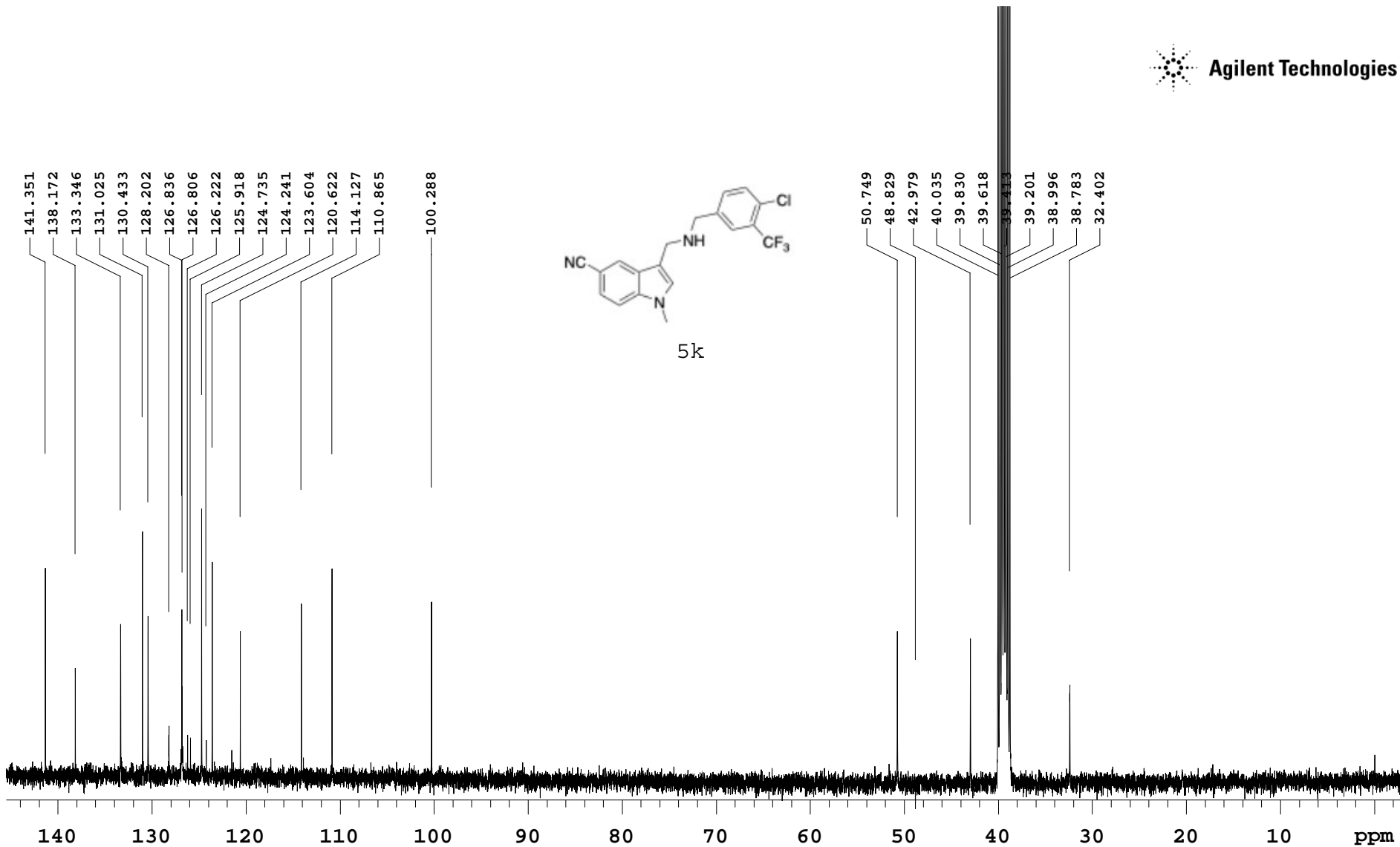

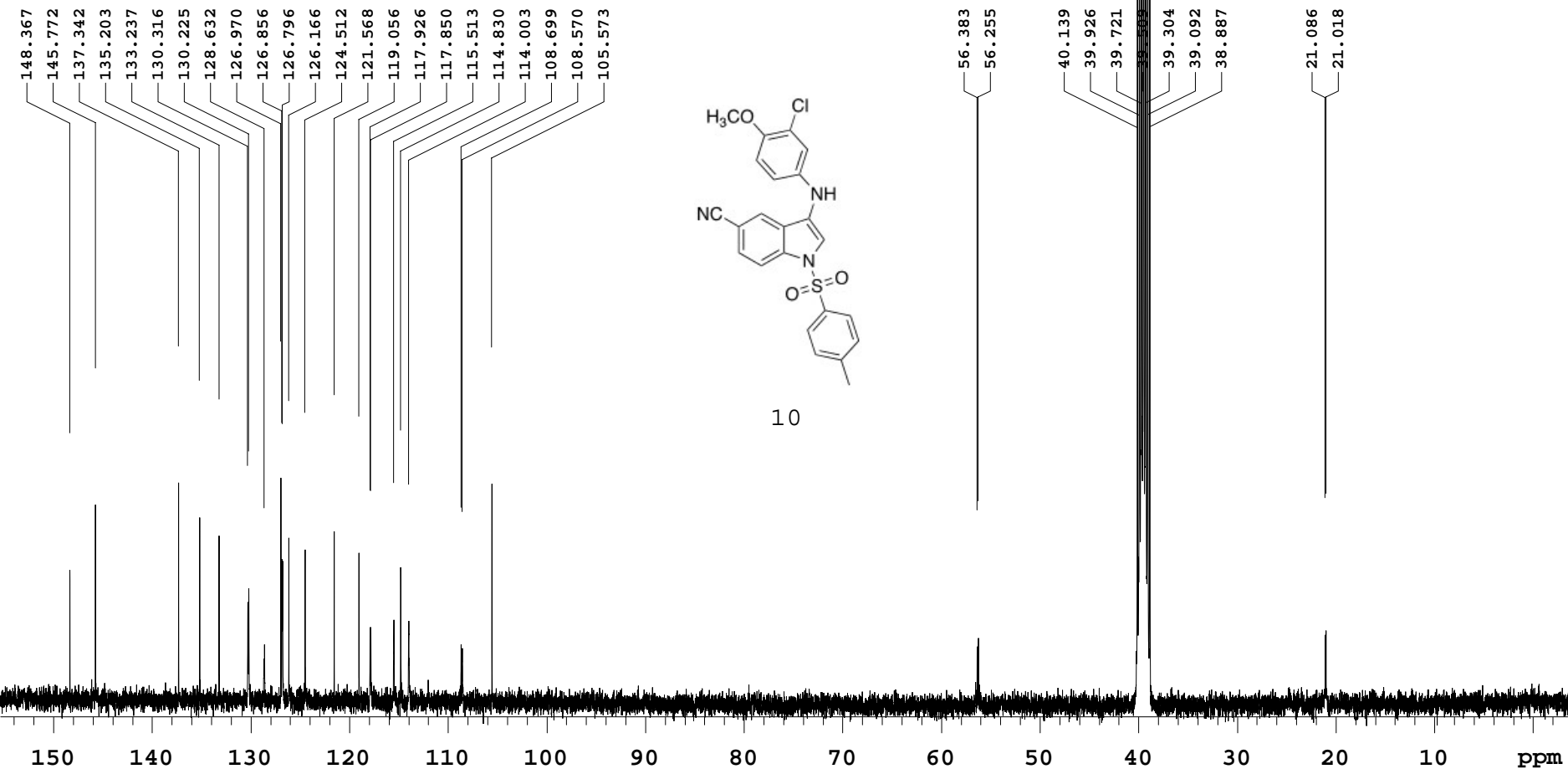

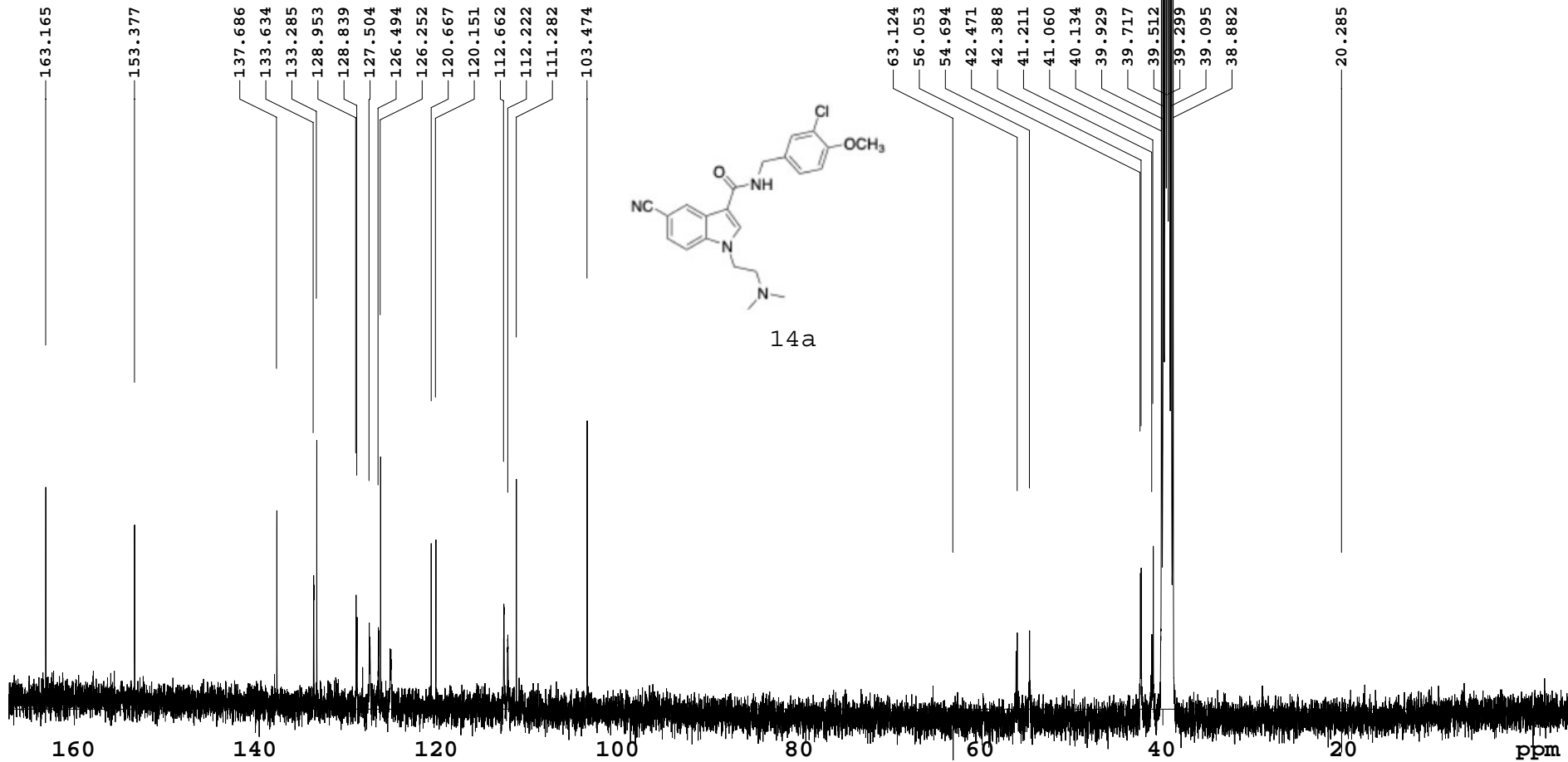

Supplement: Supplementary file 3 [file ml5c00108_si_003.pdf]
